# Fully Biobased Epoxy–Imine Monomer: Synthesis and Curing with Amines Toward Vitrimeric Materials

**DOI:** 10.1021/acssuschemeng.5c13119

**Published:** 2026-02-04

**Authors:** Pere Verdugo, Núria Montesó, Dailyn Guzman, David Santiago, Silvia De la Flor, Àngels Serra

**Affiliations:** † 303231 Eurecat, Technology Center of Catalonia, Chemical Technologies Unit, c/Marcel·lí Domingo 2, 43007 Tarragona, Spain; ‡ 16777 Universitat Rovira i Virgili, Department of Analytical and Organic Chemistry, c/Marcel·lí Domingo 1, 43007 Tarragona, Spain; § Universitat Rovira i Virgili, Department of Mechanical Engineering, Av. Països Catalans 26, 43007 Tarragona, Spain

**Keywords:** vanillin, epoxy, vitrimers, imine, sustainable, DGEBA, cystamine

## Abstract

This study presents the synthesis of a new biobased epoxy monomer derived exclusively from biobased vanillin and epichlorohydrin. Using only sustainable processes, vanillin is first converted to vanillin amine hydrochloride, which is then condensed with vanillin to obtain a diphenol containing an imine moiety. This diphenol subsequently reacts with epichlorohydrin to produce the epoxy monomer. Unlike thermosetting networks in which the imine is formed during the curing reaction, this epoxy monomer incorporates the imine moiety, and no further processing is needed after network formation due to water release. It presents a low molecular weight (399.44 g/mol), enabling the formation of a wide range of highly cross-linked materials. The epoxy monomer can be melted at 100 °C, which facilitates its formulation with the curing agents without the need for solvents. The monomer was cured in this work with two diamines, isophorone diamine (IPDA), a commonly used curing agent, and cystamine (Cyst) as a biobased amine that contains a dynamic disulfide group, to obtain a 100% biobased material. The resulting materials were compared with DGEBA/IPDA and DGEBA/Cyst formulations in terms of thermomechanical properties to evaluate whether the new monomer could be a promising alternative to DGEBA. No less important is the fact that the presence of the imine moiety in the monomer gives vitrimeric behavior to the material. This feature enables reprocessing of the material and allows for both chemical and mechanical recycling. Such properties are particularly promising for applications such as reversible adhesives or degradable matrices in composite materials.

## Graphical abstract

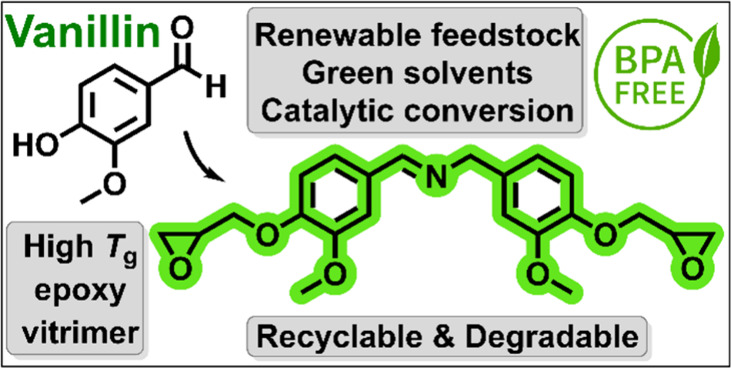

## 1 Introduction

Thermosets are a class of materials widely used in many sectors, including aerospace, electronics, and construction.^1–3^ Their cross-linked three-dimensional structure imparts excellent mechanical and thermal properties, making them highly valuable as adhesives, coatings, and matrices in composite materials. However, this same characteristic makes thermosets intrinsically difficult, or even impossible, to recycle or reprocess, due to the permanent covalent bonds in their structure.^4^ Leibler and co-workers were the first to address this problem by designing a thermosetting material containing reversible covalent bonds.^5^ They termed this new class of materials as vitrimers, named for their Arrhenius-like gradual viscosity decrease with temperature, similar to vitreous silica. This innovation allows the development of materials with the mechanical performance of thermosets while gaining the processability and recyclability of thermoplastics, thanks to their ability to rearrange their structural network.

The rearrangement of the network is achieved through the equilibration of reversible functional groups within the structure. Among them, imine bonds are widely used functional groups in vitrimer materials, as they follow associative mechanisms (transamination and imine metathesis), and show a fast dynamic exchange without the need for a catalyst.^6^ Furthermore, imines are readily hydrolyzed under acidic conditions, enabling the chemical degradation of the network at the end of the material’s service life. This degradability is particularly valuable for recovering the reinforcing fibers in composite materials, which has been already demonstrated in networks containing imine moieties.^7–9^

There are different ways to incorporate imine moieties into a polymer network. The imine can be formed at the same time as the network through polyimine synthesis,^10^ or it can be incorporated directly into the monomer^8,11^ or the curing agent.^12–14^ One drawback of the polyimine synthesis is that the condensation reaction forms water as a byproduct, generating bubbles. Consequently, the material must be hot-pressed to obtain a defect-free material.^15,16^ When the imine group is present in the monomer or curing agent, the final material can be obtained directly from the curing reaction, and the functional groups responsible for the network formation can vary (e.g., epoxy, isocyanate, etc.). Furthermore, if the dynamic group is incorporated into the monomer, the versatility of the monomer is enhanced, as every curing agent appropriate for the reactive groups would be suitable for the preparation of the network, thereby avoiding limitations to specific curing agents.

Imine moieties can be easily synthesized in high yields via the condensation reaction between an aldehyde and a primary amine.^17^ The possibility of obtaining imine groups from natural aldehydes, such as vanillin, has recently raised interest in imine-based vitrimers.^18^ It should be considered that the sustainability of materials not only has to deal with recyclability and processability but also with the raw materials used for their synthesis. Nowadays, the use of biosourced feedstock is a hot topic, driven by the depletion of fossil-based resources. Moreover, the extraction and processing of these materials cause CO_2_ emissions, further exacerbating the environmental impact of human activity. Although fossil fuels account for 80–90% of energy production, reducing their use in the chemical industry could significantly lower greenhouse gas emissions.^19^ Vanillin is nowadays available from lignin. Its phenyl and aldehyde groups allow the synthesis of a great variety of derivatives, including imines, converting vanillin into a versatile building block for material synthesis.^20^

Several studies in the literature report vanillin-based imine-containing epoxy vitrimer materials. However, most of these materials are prepared using nonrenewable amines.^11,21–27^ To the best of the authors’ knowledge, only Zhao et al. reported a phenolic imine hardener synthesized from vanillin and tyramine,^28^ which is a biobased amine. Nonetheless, tyramine is relatively expensive compared to vanillin. For this reason, preparing an imine-containing epoxy monomer with vanillin as the only starting material would be of great interest from sustainability and economic perspectives.

Inspired by the work of Zhang and co-workers,^29^ who prepared an imine-containing diol for the preparation of polyurethane foams, we envisioned the development of an epoxy monomer using vanillin and vanillin amine as starting materials. In this study, we synthesized a new biobased epoxy monomer, via a two-step conversion of vanillin into vanillin amine hydrochloride. This was followed by condensation with vanillin to form the imine, which was then subjected to glycidylation using the biobased epichlorohydrin Reodrin. Reodrin is a sustainable form of epichlorohydrin, produced from 100% biocircular, second-generation glycerin, and developed by Ineos Inovyn (Luxembourg). Its production enables greenhouse gas savings of almost 70% compared to fossil-based epichlorohydrin.^30^ Moreover, the vanillin used was also from biobased origin. In addition, all reaction steps in the synthesis of the monomer use sustainable solvents (water and ethanol) and rely on catalytic conversion to enhance the atom economy.

This monomer was cured using isophorone diamine (IPDA), a typical amine curing agent for epoxy monomers, as well as cystamine (Cyst), a biobased curing agent that incorporates disulfide as a dynamic group. While the IPDA used in this work is not derived from renewable sources, it is worth noting that Evonik has developed a range of cross-linking agents synthesized from renewable acetone with isophorone structures.^31^ Introducing disulfide groups into the curing agent creates vitrimeric materials with a dual exchange mechanism and a faster relaxation rate.^10^ The resulting materials were compared with DGEBA-based formulations to assess the potential of this new monomer as a sustainable alternative to DGEBA.

## 2 Experimental Section

### 2.1 Materials

Activated charcoal (powder, DARCO KB-G), bisphenol A diglycidyl ether (DGEBA, 170 g/epoxy equivalent), Celite (Diatomaceous earth, calcined), cystamine dihydrochloride (96%), which was previously neutralized with a 3 M sodium hydroxide solution and extracted with ethyl acetate to obtain the free base, and Palladium on carbon (Pd/C 10 wt %) were purchased from Sigma-Aldrich (St. Louis, MO, USA). Biobased (±)-epichlorohydrin (ECH, ≥ 99%, Reodrin) was purchased from Ineos Inovyn (Luxembourg). Biobased 4-hydroxy-3-methoxybenzaldehyde (Vanillin, > 96%) was purchased from Borregaard (Sarpsborg, Norway). 5-Amino-1,3,3-trimethyl cyclohexanemethylamine (isophorone diamine, (IPDA, 99%, mixture of cis and trans)) and benzyl triethylammonium chloride (BTEAC, 98%) were purchased from Acros Organics (Geel, Belgium). Hydrogen gas (>99.999%) was purchased from Linde (Dublin, Ireland). Hydrochloric acid (37%), hydroxylamine hydrochloride (99%), methanol (99.8%, extra dry), sodium acetate trihydrate (99%), and potassium bicarbonate (reagent grade) were purchased from ThermoScientific (Waltham, MA, USA). Sodium chloride was purchased from Panreac (Castellar del Vallès, Spain). Sodium hydroxide (pellets, 97%) and anhydrous magnesium sulfate (99.5%, powder) were purchased from Alfa Aesar (Haverhill, MA, USA). Absolute ethanol (EtOH, reagent grade >99.8%) and ethyl acetate (reagent grade, 99%) were purchased from VWR Chemicals (Radnor, PA, USA). Tetrahydrofuran was purchased from Honeywell. All the reagents were used as received unless otherwise specified.

### 2.2 Synthesis of 4-Hydroxy-3-methoxybenzaldoxime (Vanillyloxime, Van-Ox)

The oxime of vanillin was synthesized following a reported procedure.^32^ In a typical experiment, 18.00 g (259.03 mmol) of hydroxylamine hydrochloride and 64.40 g (473.25 mmol) of sodium acetate trihydrate were introduced into a 500 mL round-bottom flask and were solubilized into 362 mL of distilled water. Then, 36.00 g (236.61 mmol) of vanillin was suspended in the solution. The mixture was heated at 100 °C for 1 h until a clear solution was obtained. The reaction mixture was left to cool down to room temperature and the product precipitated as white crystals. The crystals were filtered, washed with cold water, and dried in a vacuum oven at 40 °C overnight, obtaining an 88% yield of the pure product. The product was characterized by NMR spectroscopy (Figure S1 and Figure S2). mp (DSC, Figure S3 = 123.8 °C).

ESI-MS, exact mass *m*/*z* [M+H^+^] = 168.0647 (Theoretical mass: 168.0655) (Figure S4).

^1^H NMR (DMSO-*d*_6_, 400 MHz, δ ppm): 10.82 (br. s, 1H, −NOH), 9.32 (br. s, 1H, Ar.–OH), 7.99 (s, 1H, CH=N), 7.16 (d, ^4^ *J* = 1.8 Hz, 1H, Ar.), 6.96 (dd, ^3^ *J* = 8.1 Hz, ^4^ *J* = 1.8 Hz, 1H, Ar.), 6.76 (d, ^3^ *J* = 8.1 Hz, 1H, Ar.), 3.77 (s, 3H, OCH_3_).

^13^C NMR (DMSO-*d*_6_, 100.6 MHz, δ ppm): 148.2 (CH=N), 148.1 (Ar.–OCH_3_), 147.9 (Ar.–OH), 124.5 (Ar.–CH=N), 120.6 (Ar.), 115.6 (Ar.), 109.3 (Ar.), 55.5 (−OCH_3_).

### 2.3 Synthesis of 4-Hydroxy-3-methoxybenzylamine Hydrochloride (Vanillin Amine Hydrochloride, Van-NH^3+^Cl^–^)

The vanillin amine hydrochloride was synthesized following a modification of a reported procedure.^32^ In a typical experiment, 9.05 g (54.14 mmol) of Van-Ox was introduced into a 2000 mL round-bottom flask and dissolved into 900 mL of absolute ethanol, once dissolved, 4.9 mL (58.68 mmol) of concentrated hydrochloric acid was added. After that, 1.80 g of Pd/C (10%) (2 g·L^–1^) was added and hydrogen gas was bubbled through the solution for 4 h at room temperature. After the complete conversion (checked by NMR) the solution was filtered off through Celite and the solvent was removed by rotary evaporation to obtain the product as a white solid in the form of 4-hydroxy-3-methoxybenzylammonium chloride (91% yield). The product was characterized by NMR spectroscopy (Figure S5 and Figure S6).

ESI-MS, exact mass *m*/*z* [M+H^+^] = 154.0862 (Theoretical mass: 154.0863) (Figure S7).

^1^H NMR (DMSO-*d*_6_, 400 MHz, δ ppm): 9.24 (br. s, 1H, −OH), 8.48 (br. s, 3H, −NH_3_^+^), 7.21 (d, ^4^ *J* = 1.6 Hz, 1H, Ar.), 6.86 (dd, ^4^ *J* = 1.6 Hz, ^3^ *J* = 8 Hz, 1H, Ar.), 6.80 (d, ^3^ *J* = 8 Hz, 1H, Ar.), 3.86 (s, 2H, CH_2_-NH_3_^+^), 3.76 (s, 3H, −OCH_3_).

^13^C NMR (DMSO-*d*_6_, 100.6 MHz, δ ppm): 147.6 (Ar.–OCH_3_), 146.9 (Ar.–OH), 124.7 (Ar. CH_2_−), 121.8 (Ar.), 115.3 (Ar.), 113.6 (Ar.), 55.8 (−OCH_3_), 42.2 (−CH_2_–NH_3_^+^).

### 2.4 Synthesis of 4-(((4-Hydroxy-3-methoxybenzyl)­imino)­methyl)-2-methoxyphenol (Divanillinimine, Van-Im)

The imine of vanillin and vanillin amine hydrochloride (Van-Im) was synthesized following a reported procedure.^33^ In a typical experiment, 10.00 g (65.72 mmol) of vanillin, 11.22 g (59.16 mmol) of vanillin amine hydrochloride, and 7.88 g (78.71 mmol) of potassium bicarbonate, solubilized into 150 mL of distilled water, were introduced into a 250 mL round-bottom flask. The mixture was stirred at room temperature overnight, and a yellow precipitate appeared afterward. The solid was filtered and thoroughly washed first with distilled water and then with ethyl acetate. The product was dried in a vacuum oven at 60 °C overnight, obtaining a 76% yield of the pure product. The product was characterized by NMR spectroscopy (Figure S8 to Figure S12). mp (DSC, Figure S13) = 173.0 °C.

ESI-MS, exact mass *m*/*z* [M+H^+^] = 288.1219 (Theoretical mass: 288.1230) (Figure S14).

^1^H NMR (DMSO-*d*_6_, 400 MHz, TMS, δ ppm): 9.15 (br. s, 2H, −OH), 8.27 (s, 1H, CH=N−), 7.36 (d, ^4^ *J* = 2 Hz, 1H, Ar.), 7.15 (dd, ^4^ *J* = 2 Hz, ^3^ *J* = 8 Hz, 1H, Ar.), 6.88 (d, ^4^ *J* = 2 Hz, 1H, Ar.), 6.85 (d, ^3^ *J* = 8 Hz, 1H, Ar.), 6.76 (d, ^3^ *J* = 8 Hz, 1H, Ar.), 6.70 (dd, ^4^ *J* = 2 Hz, ^3^ *J* = 8 Hz, Ar.), 4.59 (s, 2H, CH_2_–NH_2_), 3.78 (s, 3H, −OCH_3_), 3.75 (s, 3H, −OCH_3_).

^13^C NMR (DMSO-*d*_6_, 100.6 MHz, δ ppm): 160.9 (CH=N−), 149.7 (Ar.–OH), 148.1 (Ar.–OCH_3_), 147.6 (Ar.–OCH_3_), 145.5 (Ar.–OH), 130.8 (Ar.–CH_2_−), 127.9 (Ar.–CH=N−), 123.1 (Ar.), 120.7 (Ar.), 115.5 (Ar.), 115.4 (Ar.), 112.5 (Ar.), 110.0 (Ar.), 63.9 (−CH_2_–N=), 55.7 (−OCH_3_), 55.6 (−OCH_3_).

### 2.5 Synthesis of N-(3-Methoxy-4-(oxiran-2-ylmethoxy)­benzyl)-1-(3-methoxy-4-(oxiran-2-ylmethoxy)­phenyl)­methanimine (Diglydidyl Divanillinimine, Gly-Van-Im)

The diglycidyl divanillinimine (Gly-Van-Im) was synthesized following a modification of a reported procedure for the synthesis of imine-containing epoxy monomers.^34^ In a typical experiment, 23.79 g (82.80 mmol) of Van-Im, 1.91 g (8.39 mmol) of benzyl triethylammonium chloride (BTEAC), and 65.0 mL (829.01 mmol) of epichlorohydrin were introduced into a 250 mL two-necked round-bottom flask. The mixture was stirred at 80 °C for 1 h, then the mixture was cooled down in an ice–water bath and 7.36 g (184.00 mmol) of sodium hydroxide solubilized in 29.14 g of anhydrous methanol, was added dropwise. The mixture was stirred for an additional 4 h, and then the mixture was diluted with 500 mL of ethyl acetate and washed thrice with a sodium chloride solution. The organic phase was dried over anhydrous magnesium sulfate and the solvent was eliminated under reduced pressure. The product was then solubilized in 800 mL of hot ethanol, treated with activated charcoal, filtered, and recrystallized at 4 °C overnight. The product, crystallized as a white solid, was filtered, washed with cold ethanol, and dried in a vacuum oven at 40 °C overnight. A 41% yield of the pure product was obtained. The product was characterized by NMR spectroscopy (Figure 1 and Figures S15 to S17). mp (DSC, Figure S18) = 94.8 °C

**Figure 1:**
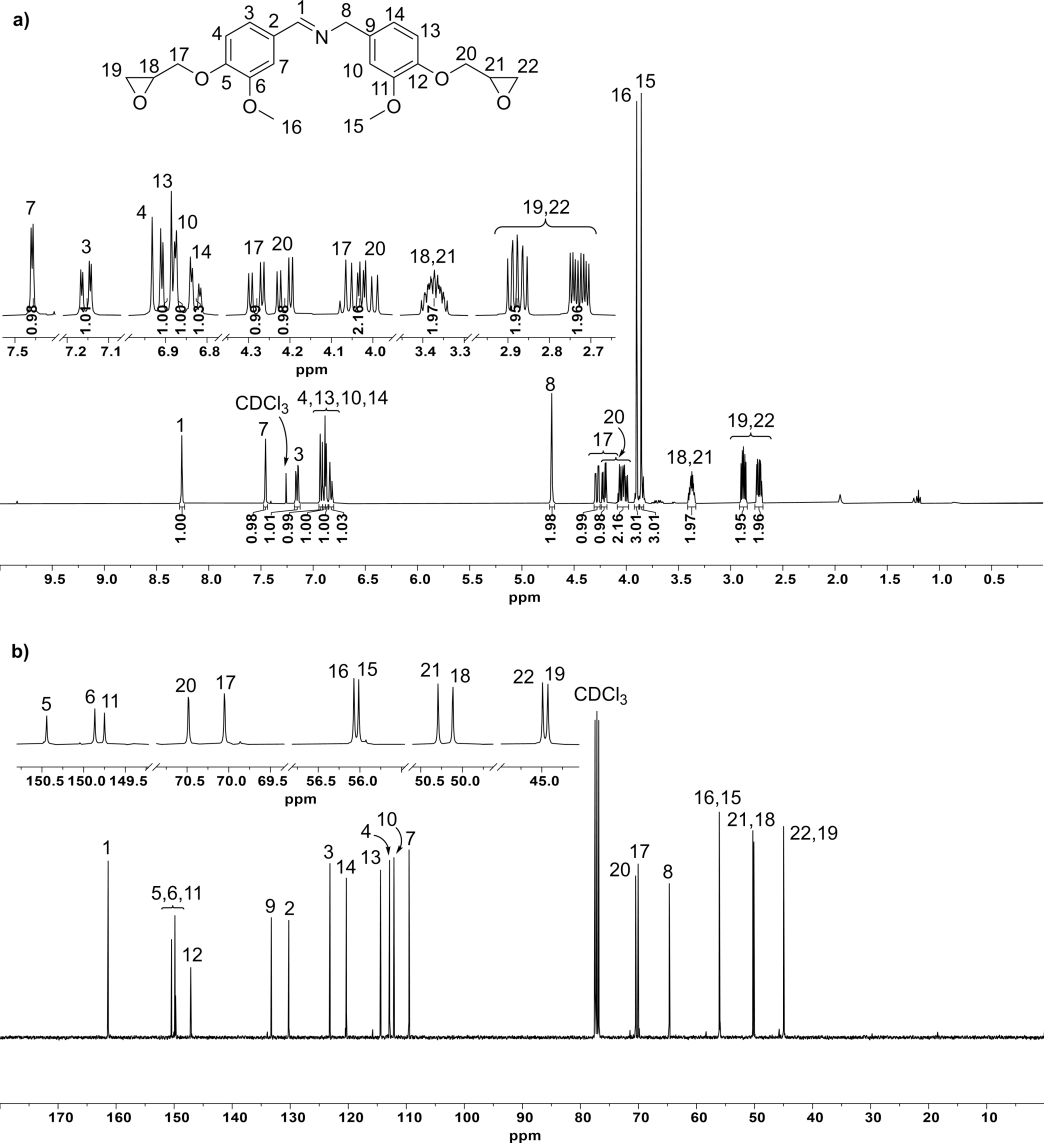
(a) ^1^H NMR and (b) ^13^C NMR spectra in CDCl_3_ of the Gly-Van-Im monomer after recrystallization.

ESI-MS, exact mass *m*/*z* [M+H^+^] = 400.1737 (Theoretical mass: 400.1755) (Figure S19).

^1^H NMR (CDCl_3_, 400 MHz, δ ppm): 8.26 (s, 1H, N=CH−), 7.46 (d, ^4^ *J* = 2 Hz, 1H, Ar.), 7.15 (dd, ^4^ *J* = 2 Hz, ^3^ *J* = 8 Hz, 1H, Ar.), 6.92 (d, ^3^ *J* = 8 Hz, 1H, Ar.), 6.90 (d, ^3^ *J* = 8 Hz, 1H, Ar.), 6.88 (d, ^4^ *J* = 2 Hz, 1H, Ar.), 6.83 (dd, ^4^ *J* = 2 Hz, ^3^ *J* = 8 Hz, 1H, Ar.), 4.71 (s, 2H, =N–CH_2_–Ar.), 4.28 (dd, ^2^ *J* = 12 Hz, ^3^ *J* = 4 Hz, 1H, CH_2_O–Ar.), 4.21 (dd, ^2^ *J* = 12 Hz, ^3^ *J* = 4 Hz, 1H, CH_2_O–Ar.), 4.03 (m, 2H, CH_2_O–Ar.), 3.90 (s, 3H, OCH_3_), 3.86 (s, 3H, OCH_3_), 3.40–3.34 (m, 2H, CH-CH_2_O), 2.90–2.85 (m, 2H, CH_2_O), 2.75–2.71 (m, 2H, CH_2_O).

^13^C NMR (CDCl_3_, 100.6 MHz, δ ppm): 161.4 (N=CH−), 150.4 (Ar.–O–Gly), 149.9 (Ar.–OCH_3_), 149.8 (Ar.–OCH_3_), 147.1 (Ar.–O-Gly), 133.3 (Ar.–CH_2_−), 130.2 (Ar.–CH=N−), 123.2 (Ar.), 120.3 (Ar.), 114.4 (Ar.), 112.9 (Ar.), 112.1 (Ar.), 109.5 (Ar.), 70.5 (CH_2_O–Ar.), 70.1 (CH_2_O–Ar.), 64.7 (=N–CH_2_−), 56.1 (−OCH_3_), 56.0 (−OCH_3_), 50.3 (CH–CH_2_O), 50.1 (CH–CH_2_O), 45.0 (CH_2_O), 44.9 (CH_2_O).

### 2.6 Preparation of the Formulations

The synthesized Gly-Van-Im was formulated with isophorone diamine (IPDA) or cystamine (Cyst) and compared with the formulation of DGEBA and Cyst (Scheme 1). All the formulations were prepared in stoichiometric proportions of epoxide:amine groups (2:1 mol:mol). The composition of the formulations prepared is detailed in Table 1. As a typical sample preparation, 2.5082 g (6.28 mmol) of Gly-Van-Im was weighed into a 20 mL vial and was melted at 100 °C. Once melted, the vial was removed from the thermostatic bath and 0.5444 g (3.20 mmol) of IPDA were added. The mixture was homogenized with manual stirring using a spatula and poured into Teflon molds. The formulation was cured in an oven 2 h at 120 °C, 2 h at 140 °C and 1 h at 160 °C. Samples were polished with sandpaper until obtaining the desired dimensions.

**Scheme 1:**
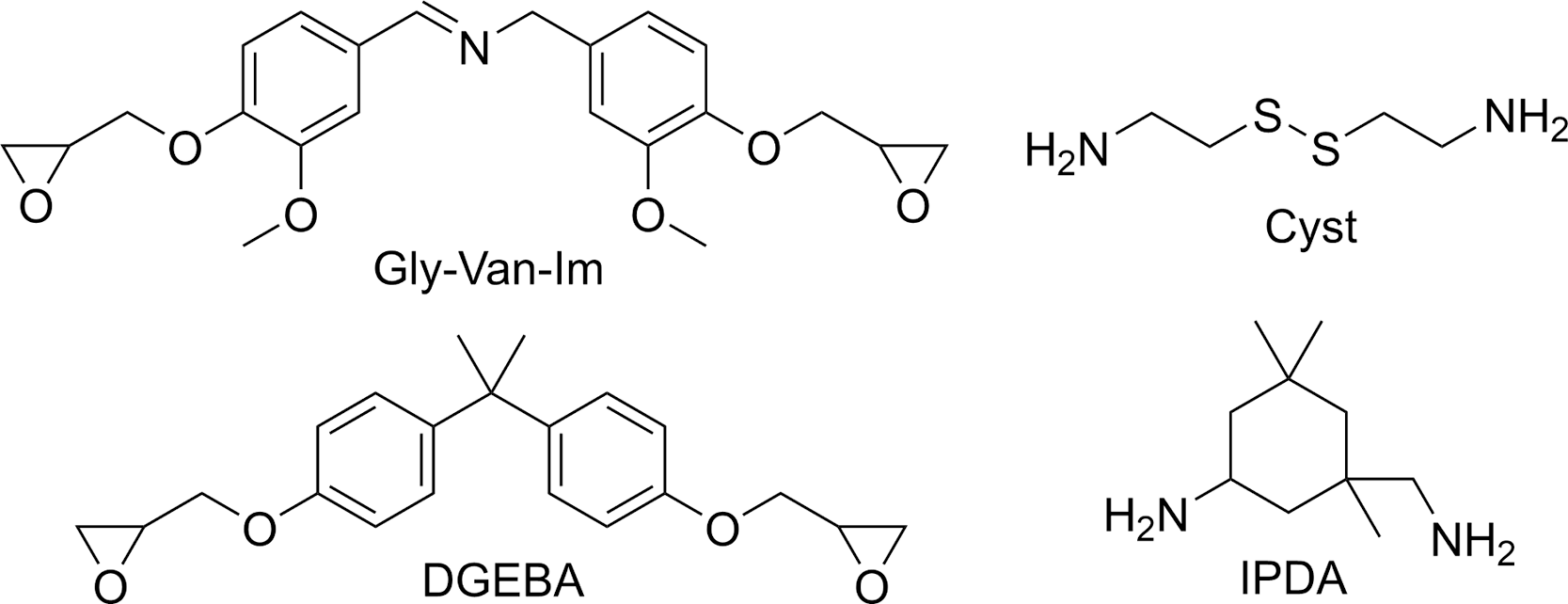
Chemical Structures of the Vanillin-Based Epoxy Monomer (GlyVan-Im), Diglycidyl Ether of Bisphenol A (DGEBA), Cystamine (Cyst), and Isophorone Diamine (IPDA)

**Table 1:** Monomer/Curing Agent Weight Proportions in the Prepared Formulations

Formulation	Monomer (g)	Amine (g)
Gly-Van-Im/IPDA	2.5082	0.5444
Gly-Van-Im/Cystamine	2.4070	0.4641
DGEBA/IPDA	2.5357	0.6392
DGEBA/Cystamine	2.4769	0.5576

### 2.7 Monomer Characterization

All the synthesized products and the products obtained after the chemical degradation were characterized by NMR spectroscopy (^1^H NMR and ^13^C NMR) using a Varian VNMR-S400 NMR spectrometer (Agilent Technologies, Santa Clara, CA, USA). CDCl_3_ and DMSO-*d*_6_ were used as the solvents. All chemical shifts were quoted on the δ scale in part per million (ppm) using the residual solvent peak as reference (^1^H NMR: CDCl_3_ = 7.26 ppm, DMSO-*d*_6_ = 2.50 ppm, and ^13^C NMR: CDCl_3_ = 77.16 ppm, DMSO-*d*_6_ = 39.52 ppm). The exact mass of the synthesized products was analyzed on a Thermo Scientific Orbitrap IDX Tribrid mass spectrometer with a HESI interface, in line with Vanquish UHPLC Liquid Chromatograph. No column was used. ACN/H_2_O (A) and MeOH/formic acid 0.1% (B) at 50% each were used for elution. The flow was set to 0.15 mL/min, respectively. An injection volume of 1 mL was used.

### 2.8 Thermal Characterization

The study of the curing process was performed by differential scanning calorimetry (DSC) using a Mettler-Toledo DSC3+ (Columbus, OH, USA) instrument calibrated using indium (heat flow calibration) and zinc (temperature calibration) standards. Samples of approximately 8–10 mg were placed in aluminum pans with pierced lids and analyzed under a flow of N_2_ at 50 mL·min^–1^. The curing process was studied in nonisothermal mode at 10 °C·min^–1^ from 30 to 250 °C. The glass transition temperature (*T*_*g*_) of the cured samples was determined in dynamic scans at 50 °C·min^–1^ from −20 to 180 °C.

The thermal stability of cured samples was evaluated by thermogravimetric analysis (TGA), using a Mettler-Toledo TGA 2 thermobalance (Columbus, OH, USA). All the experiments were performed under a flow of N_2_ at 50 mL·min^–1^. Pieces of cured samples of a mass of approximately 10 mg were degraded between 30 and 600 °C at a heating rate of 10 °C·min^–1^. The thermal stability was also studied in isothermal mode at 180 °C for 3 h.

### 2.9 Thermomechanical Characterization

Thermomechanical properties were measured using a TA Instruments Discovery DMA 850 (New Castle, DE, USA) equipped with a tension film clamp. Prismatic rectangular samples of about 30 mm × 6 mm × 1.5 mm were analyzed at 1 Hz, 0.1% strain, and from 0 to 220 °C at 3 °C·min^–1^. The storage modulus at glassy state (*E*′_*g*_) and at rubbery state (*E*′_*r*_) were obtained at *T*_*g*_ - 50 °C and at *T*_*g*_ + 50 °C, respectively. The *T*_*g*_s were determined from the maximum of the peak of tanδ.

### 2.10 Stress Relaxation Tests

Stress relaxation tests were carried out using a TA Instruments Discovery DMA 850 (New Castle, DE, USA) equipped with a film tension clamp on samples with the same dimensions as previously defined. Samples were first equilibrated at temperatures slightly above the *T*_*g*_ of each formulation, then a constant strain of 1% (within the linear range) was applied to the sample, and the consequent stress level was monitored as a function of time. The process was repeated every 10 °C, up to 160 °C, depending on the formulation.

The stress σ was normalized by the initial stress *σ*_*0*_, and this relative stress were fitted to the Kohlrausch–Williams–Watts (KWW) stretched exponential decay function (eq 1) in the case of formulations Gly-Van-Im/IPDA and DGEBA-Cyst.

1 $$\frac{\sigma }{\sigma_{0}}=\exp{\left(\frac{-t}{\tau }\right)}^{\beta }$$

The KWW model is used to describe the stress relaxation process of vitrimers, and polymers in general, considering the distribution of overlapping relaxation modes that contribute to the macroscopic stress relaxation of the material. The breadth of the relaxation distribution is described by the stretching parameter β, τ is the characteristic relaxation time and *t* is the decay time.

A multimode Maxwell model was used to describe the behavior of Gly-Van-Imp (eq 2).

2 $$\frac{\sigma }{\sigma_{0}}=A\exp\left(\frac{-t}{\tau_{1}}\right)+\left(1-A\right)\exp\left(\frac{-t}{\tau_{2}}\right)$$

The equation determines two characteristic relaxation times *τ*_*1*_ and *τ*_*2*_, related to two different Maxwell elements, *A* is the pre-exponential factor, and *t* is the decay time.

The activation energy *E*_*a*_ was calculated for each material by using an Arrhenius-type eq (eq 3):

3 $$\ln\tau =\frac{E_{a}}{RT}-\ln A$$

where τ is the characteristic relaxation time, *R* is the gas constant, *T* is the absolute temperature, and *A* is the pre-exponential factor.

### 2.11 Creep Experiments

Creep and recovery properties were studied using a TA Instruments Discovery DMA 850 (New Castle, DE, USA) equipped with a film tension clamp with the same dimensions as previously defined. A stress of 0.1 MPa was applied for 10 min at 110 °C, then the stress was immediately released, and the sample was left to recover for another 30 min. This procedure was repeated every 10 °C, up to 190 °C, depending on the formulation. The viscosity η was calculated using eq 4:

4 $$\eta =\frac{\sigma }{\mathrm{\varepsilon ̇}}$$

The deformation rate was determined as the slope of the linear fit of the linear part of the variation of the strain as a function of time. The Angell Fragility plot was then obtained by plotting η as a function of *T*_*g*_·*T* ^–1^, and the topology freezing temperature *T*_*v*_ was determined as the temperature at which the material reaches a viscosity of 10^12^ Pa·s.

## 3 Results and Discussion

### 3.1 Synthesis of Diglycidyl Divanillin Imine Monomer (Gly-Van-Im)

The synthesis of the Gly-Van-Im monomer was carried out in a four-step protocol, beginning with the formation of vanillyl oxime, followed by its reduction to vanillin amine hydrochloride. The imine was then formed via the condensation of vanillin amine hydrochloride with vanillin, and finally, glycidyl moieties were introduced by reaction with epichlorohydrin (Scheme 2). All steps involved in the monomer synthesis were performed using sustainable solvents and environmentally friendly reaction conditions.

**Scheme 2:**
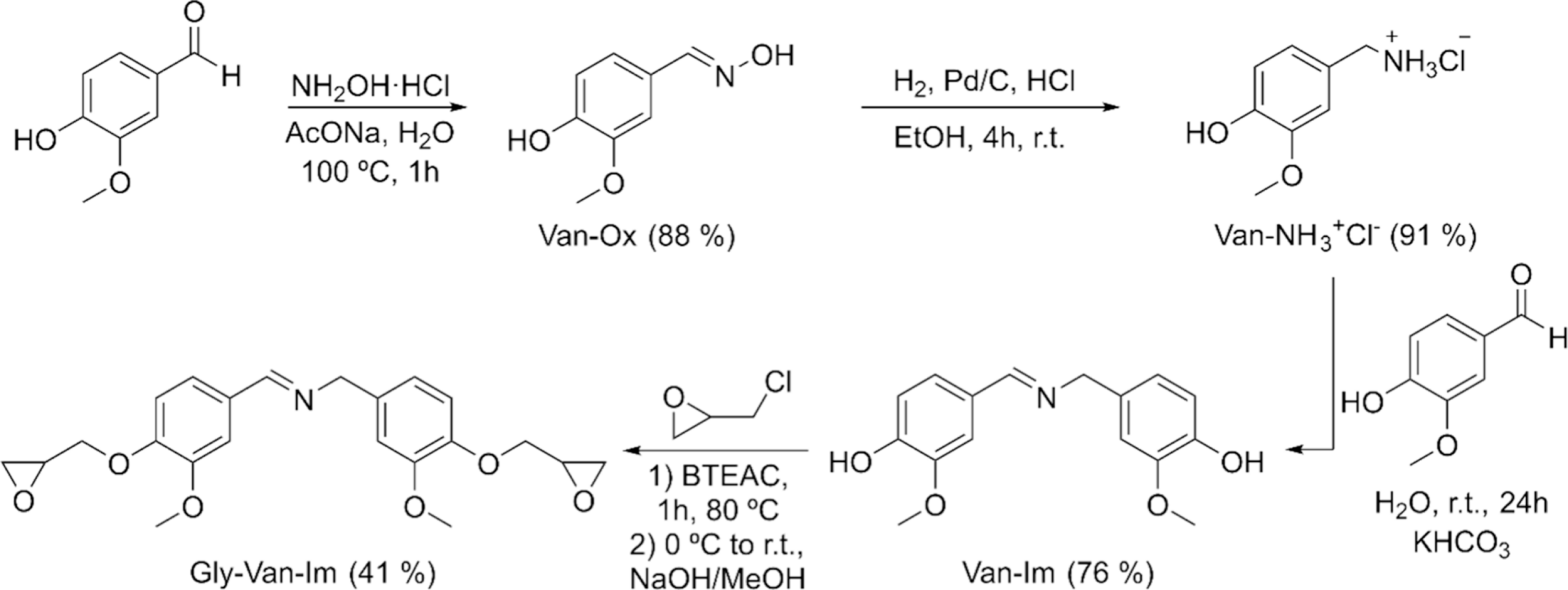
Synthesis of Diglycidyl Divanillin Imine (Gly-Van-Im) in a Four-Step Protocol

The first step involves the preparation of vanillyl oxime (Van-Ox) by the condensation reaction of vanillin with hydroxylamine, following the procedure reported by Fache et al.^32^ Oximes are commonly used as intermediates to obtain amines through catalytic hydrogenation,^35,36^ and are easily synthesized by the condensation of aldehydes with hydroxylamine.

The reaction was performed in distilled water at 100 °C for 1 h and gave white crystals on cooling, which were filtered and washed with cold water, yielding 88% of the pure product. This procedure offers a simple and efficient route to pure Van-Ox derivative. The product was completely characterized by NMR spectroscopy (Figure S1 and Figure S2), ESI-MS (Figure S4), and the melting point determined by DSC (Figure S3). This first intermediate was reduced to the amine by catalytic hydrogenation with Pd/C. The catalytic reduction of oximes can lead to different products depending on the catalyst used, oximes can evolve to hydroxylamines (using Pt/C) or to amines (Pd/C).^36^ Additionally, strong acids (e.g., H_2_SO_4_) can induce a Beckmann rearrangement to amides, or oxime dehydration to nitriles, which hydrogenate to imines. Imine must be rapidly hydrogenated because they can react with primary amines to produce secondary amines (Scheme 3).^37^ To avoid the formation of secondary amines, the reactions are often run in the presence of hydrochloric acid, converting amines to inert hydrochloride salts.

**Scheme 3:**
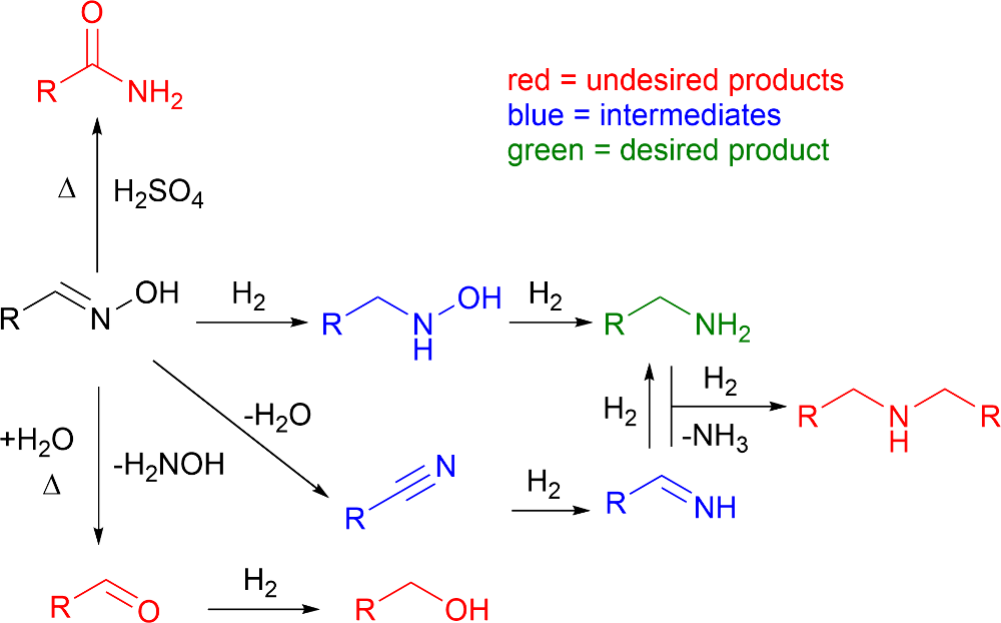
Possible Side Reactions in the Hydrogenation of Aldoximes, Adapted from Ref 34

The procedure followed was a modification of that reported by Fache.^32^ The Van-Ox solubilized in absolute ethanol (10 g·L^–1^) was treated with 1.0 equiv of concentrated HCl. After that, Pd/C (10 wt %) was added at a concentration of 2 g·L^–1^, and H_2_ was bubbled for 4 h. To obtain the pure Van-NH_3_^+^Cl^–^, the crude product was filtered through Celite to remove the Pd/C, and the solvent was evaporated under reduced pressure. The pure product was characterized by NMR spectroscopy (Figure S5 and Figure S6) and ESI-MS (Figure S7).

The next step was the condensation reaction between vanillin and vanillin amine with the release of water, which is typically eliminated to shift the equilibrium toward the imine. One common strategy to drive the reaction forward involves using a solvent in which the product has low solubility, allowing it to precipitate as it forms. A typical reaction medium for this approach is ethanol. By performing the reaction at reflux overnight, the imine precipitated from the ethanolic solution as a solid, which only needs to be filtered and washed with cold ethanol to obtain the pure product.^34^ Since in our case the amine was in its hydrochloride form, it would have had to be neutralized and recovered before the condensation reaction in ethanol, which could have led to product losses and therefore would have been detrimental to the overall yield. Wu P. H. et al.^29^ reported a more straightforward and sustainable method for preparing the imine derivative without requiring prior neutralization of the amine salt and using water as the reaction medium. Following their procedure, we solubilized the vanillin amine hydrochloride and vanillin in water and used potassium bicarbonate for the in situ neutralization of the salt. The reaction was carried out at room temperature for 12 h, after which the product precipitated from the reaction medium. The solid was collected by filtration and washed with water and ethyl acetate, obtaining excellent yields. The product obtained was fully characterized by NMR spectroscopy (Figure S8 to Figure S12), ESI-MS (Figure S14), and the melting point determined by DSC (Figure S13).

The final monomer (Gly-Van-Im) was obtained via glycidylation with epichlorohydrin in the presence of benzyl triethylammonium chloride as a phase-transfer catalyst. Sodium hydroxide in methanol was added to promote oxirane formation while minimizing imine hydrolysis.^34^

The obtained product showed only a minimal amount of the hydrolyzed byproduct, which was determined by ^1^H NMR spectroscopy of the crude product after solvent evaporation (Figure S20). By comparing the intensity of aldehyde (−CHO) and imine (CH = N) signals, the proportion of hydrolyzed product was estimated to be around 7 mol %. Moreover, some unreacted epichlorohydrin (ECH) was observed in the crude product. After extensive drying under high vacuum at 40 °C overnight to eliminate any remaining epichlorohydrin, the obtained product was a viscous oil, probably due to the presence of impurities. These impurities may be monoglycidyl ether of vanillin and the triglycidyl ether of vanillin amine. Their presence could lead to the formation of permanent bonds in the final network, and although this effect would be minimal, for reproducibility purposes, the monomer was purified. For purification, the crude monomer was solubilized in hot ethanol, treated with activated charcoal, filtered, and the solution cooled to room temperature and then stored at 4 °C overnight, yielding a 41% of a white solid (mp 94.8 °C, Figure S18) with a clean ^1^H- and ^13^C NMR spectra (Figure 1).

The purified product was fully characterized by NMR spectroscopy, including bidimensional spectra (Figure S15 to Figure S17), and the exact mass determined by ESI-MS (Figure S19) matched the theoretical value.

The overall yield of the synthesis is 25% over four steps, which is a commendable outcome given the high purity of the final monomer. Moreover, the entire process employs sustainable solvents such as water and ethanol, along with inexpensive reagents, highlighting the efficiency and environmental friendliness of the methodology.

### 3.2 Study of the Curing Process

To investigate the curing process, four formulations were prepared: two with the synthesized monomer and two with DGEBA, for comparative purposes. Cystamine (Cyst), a disulfide-containing diamine, was selected to prepare fully biobased materials and to enhance vitrimeric behavior by the combination of two exchange mechanisms, imine metathesis and disulfide exchange.

The acceleration of stress relaxation by the use of dual relaxation mechanisms has been already proved in our group^8,38^ and others.^39^ Isophorone diamine (IPDA), a commonly used amine curing agent, was selected to compare the thermal properties with those of the fully biobased formulation.

The curing of all formulations was studied by differential scanning calorimetry (DSC). Figure 2 shows the overlaid thermograms, and the corresponding data are collected in Table 2. Both DGEBA/IPDA and DGEBA/Cyst formulations present higher exothermic peaks at higher temperatures (123 °C) than Gly-Van-Im formulations (102 °C), reflecting differences in oxirane reactivity due to stereoelectronic effects.^40^

**Figure 2:**
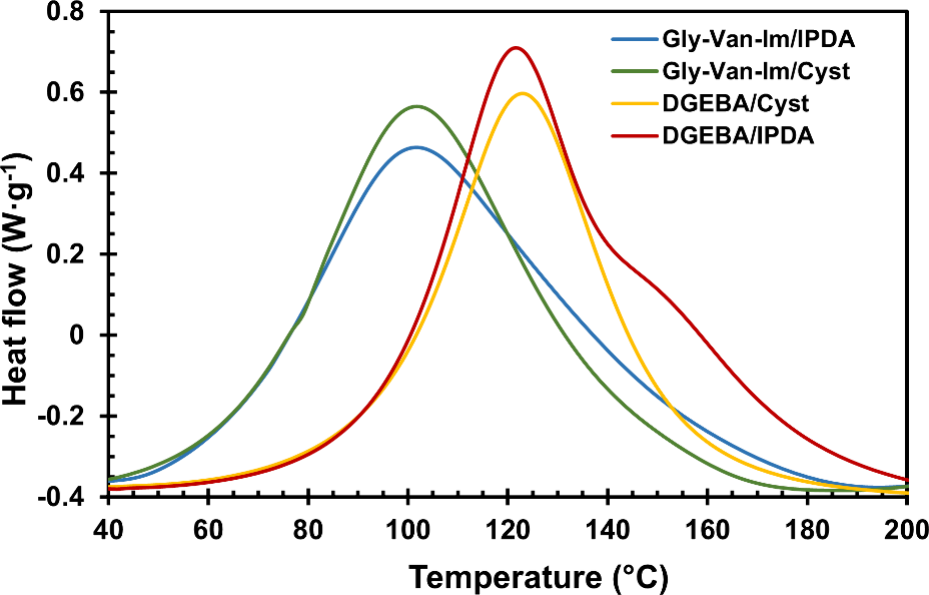
DSC curing exotherms for the prepared formulations.

**Table 2:** Calorimetric Data of the Curing Process and Thermal Stability Data of All the Studied Formulations in an N_2_ Atmosphere

Formulation	*T*_peak_ ^a^ (°C)	Δ*H* ^b^ (J·g^–1^)	Δ*H* ^c^ (kJ·ee^–1^)	*T*_g_ ^d^ (°C)	*T*_2%_ ^e^ (°C)	*T*_max_ ^f^ (°C)	Char yield^g^ (%)
Gly-Van-Im/IPDA	102	298	73	128	275	328	30
Gly-Van-Im/Cyst	102	366	87	96	221	320	37
DGEBA/IPDA	122	451	96	160	336	374	10
DGEBA/Cyst	123	482	101	111	251	265/352	10

a Temperature of the maximum of the exotherm of the epoxy-amine reaction.

b Enthalpy released during curing by gram.

c Enthalpy released by epoxy equivalent.

d Glass transition temperature of the final cured material determined by DSC.

e Temperature of 2% of weight loss in N_2_.

f Temperatures at the maximum rate of degradation.

g Char residue at 600 °C.

The DGEBA/IPDA formulation presents a shoulder at higher temperatures, which can be attributed to the lower reactivity of the amine linked with the secondary carbon in IPDA, as previously reported in the literature.^41^ A similar effect is observed in the Gly-Van-Im/IPDA formulation, which displays a broader curing exotherm compared to the Gly-Van-Im/Cyst formulation. This observation is consistent with the reduced reactivity of the amine linked to the secondary carbon in IPDA.

The typical heat for the epoxy/amine reaction is approximately 100 kJ·mol^–1^ of epoxy group,^42^ consistent with DGEBA formulations (Table 2). In contrast, Gly-Van-Im formulations show slightly lower curing enthalpy. This can be attributed to partial curing of the biobased formulations prior to DSC analysis, as a result of the higher reactivity of the Gly-Van-Im monomer and the need to prepare the formulations at 100 °C, due to the monomer’s melting point. Nevertheless, the cured material was analyzed by DSC, showing only a *T*_g_ and no evidence of residual heat (Figure S21), confirming that the material was completely cured.

### 3.3 Thermal Characterization

The thermal stability of the materials was evaluated by thermogravimetric analysis (TGA). Figure 3a shows the TGA curves for each formulation, while Figure 3b presents the first derivative of the TGA of these curves (DTG). Degradation data are summarized in Table 2.

**Figure 3:**
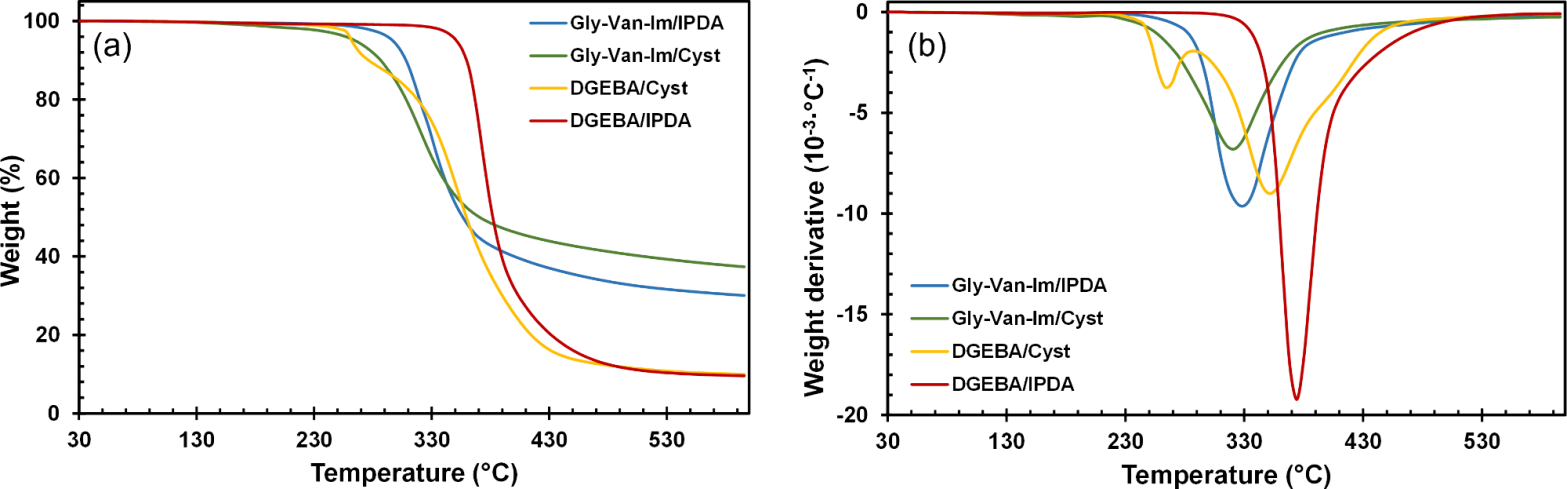
(a) TGA curves and (b) DTG curves for all the prepared materials.

DGEBA/IPDA sample exhibits the highest thermal stability, as expected due to the absence of labile bonds, with a temperature of 2% weight loss (*T*_2%_) at 336 °C, considerably higher than in the other materials. In the first derivative, we can observe only one peak, indicating one degradation mechanism with a maximum rate of degradation at 374 °C. In contrast, the DGEBA/Cyst formulation presents lower thermal stability (*T*_2%_ of 251 °C) with two DTG peaks: the first with a maximum at 265 °C, likely from the disulfide cleavage, as it is the most labile bond and the second, at 352 °C from the polymer backbone degradation.^43^ This is closer in temperature to the degradation of the DGEBA/IPDA material. However, the char yield appears to be unaffected by the type of curing agent, as both DGEBA-based formulations exhibited a residual mass of approximately 10%. Gly-Van-Im/IPDA material exhibits a *T*_2%_ of 275 °C, about 60 °C lower than DGEBA/IPDA material, highlighting the imines impact on the reduced thermal stability.^44^ The derivative curves of both materials show single peaks (*T*_max_) at 328 and 374 °C, respectively. The Gly-Van-Im/Cyst material presents the lower stability among all the materials studied, with a *T*_2%_ of 221 °C. It is worth mentioning that the char yields after degradation at 600 °C are high (30 and 37%) for these materials, due to their high nitrogen content and their aromatic structure, which could provide good flame-retardant properties. The imine flame-retardant behavior is attributed to the formation of a nitrogen-containing hexatomic ring during combustion.^45^

To check their stability under potential reprocessing conditions, all materials were subjected to isothermal TGA experiments at 180 °C for 3 h (Figure S22). The DGEBA/IPDA formulation shows the lowest mass loss, with a 0.4%, while DGEBA/Cyst, Gly-Van-Im/IPDA, and Gly-Van-Im/Cyst showed mass losses of 0.8, 0.9, and 1.7%, respectively. These results clearly indicate that the disulfide group makes a major contribution to the loss of thermal stability. Nevertheless, all materials demonstrate acceptable long-term thermal stability under moderate reprocessing conditions.

### 3.4 Thermomechanical Characterization

Figure 4 shows the evolution of the storage modulus (*E*′, Figure 4a) and the damping factor (tan δ, Figure 4b) with the temperature for the studied materials. The main thermomechanical data obtained from these experiments are listed in Table 3. All materials showed relatively high glass transition temperatures (*T*_g_s), ranging from 99 °C up to 167 °C. When cured with cystamine, the *T*_g_ values were 99 °C for Gly-Van-Im and 109 °C for DGEBA. When IPDA was the curing agent, the *T*_g_s increased to 128 °C for Gly-Van-Im and 167 °C for DGEBA. This trend is expected considering that cystamine is an aliphatic and flexible curing agent, while IPDA leads to tighter and more rigid network structure. Despite the structural similarities between Gly-Van-Im and DGEBA monomers, the *T*_*g*_ values differ considerably when cured with the same hardener. The structural factors may account for this behavior. First, the structure Gly-Van-Im has two methoxy pendant groups that, although relatively small, can limit the polymer chains stacking, reduce intermolecular interactions, and thus impart greater mobility to the network.^46,47^ In Gly-Van-Im, the imine group introduces a longer spacer between aromatic rings than the isopropylidene bridge in DGEBA. Although the imine bond locally restricts motion, the longer backbone increases flexibility, giving the network greater segmental mobility than in DGEBA.

**Figure 4:**
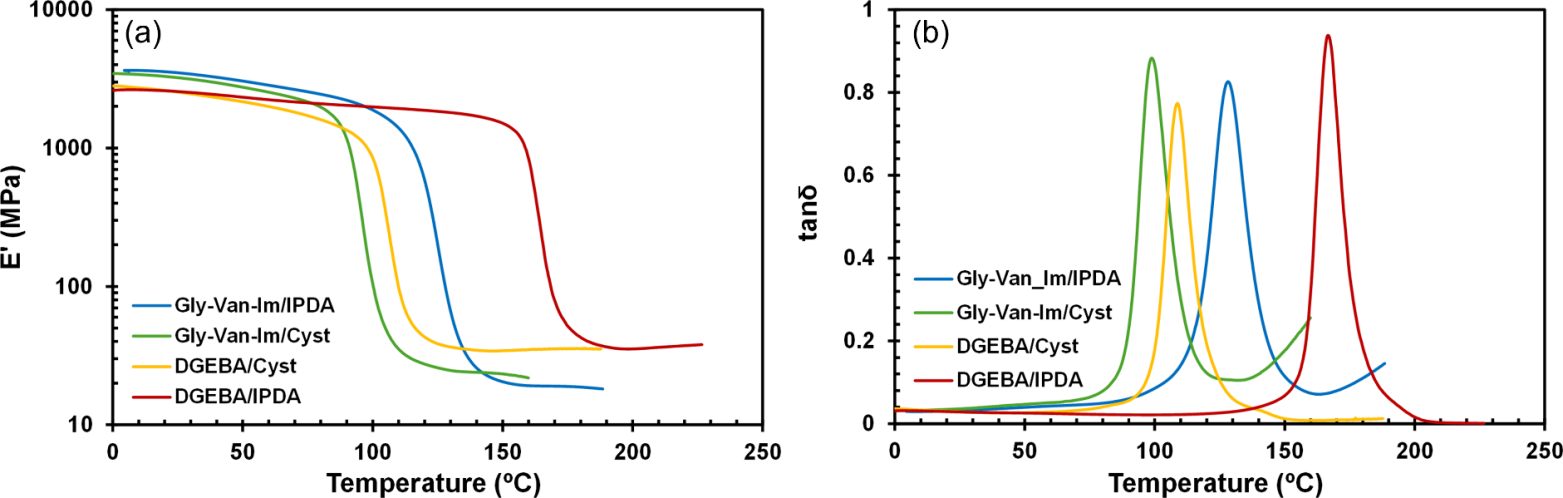
(a) Storage modulus E′ (a) and tan δ (b) as a function of temperature for the prepared materials.

**Table 3:** Thermomechanical Data of the Prepared Thermosetting Polymers

Material	*T*_*tanδ*_ ^a^ (°C)	fwhm^b^ (°C)	*E*′_*g*_ ^c^ (MPa)	*E*′_*r*_ ^d^ (MPa)
Gly-Van-Im/IPDA	128	17	2492	19
Gly-Van-Im/Cyst	99	13	2778	23
DGEBA/IPDA	167	12	1895	37
DGEBA/Cyst	109	12	2013	35

a Temperature of the maximum of the tan δ peak.

b Full width at half-maximum.

c Storage modulus measured at *T*_*g*_ – 50 °C;

d Storage modulus measured at *T*_*g*_ + 50 °C.

These factors explain the *T*_g_ differences between the DGEBA and Gly-Van-Im based materials, which are more pronounced when IPDA was used as the curing agent (39 °C difference) than with cystamine (10 °C). This suggests that the high flexibility of cystamine governs network dynamics, largely masking structural differences between the monomers. The values of storage modulus in the rubbery region (*E*′_*r*_, Table 2) suggest that DGEBA-based materials have a higher cross-linking density, which ultimately leads to their higher *T*_g_s. The values of *E*′_*r*_ can be correlated with the cross-linked density of thermosets.^48^ In this case, the trend in *E*′_*r*_ can be explained by the longer distance between glycidyl groups in Gly-Van-Im. Interestingly, in the glassy region, the *E*′_*g*_ values are higher in materials derived from Gly-Van-Im. According to Hernandez et al.,^46^ the oxygen atoms in methoxy group participate in hydrogen bonding with the hydroxyl groups formed in the epoxy–amine reaction, resulting in greater rigidity at low temperatures. The presence of the imine group can also lead to the formation of hydrogen bonds.

All materials exhibit good homogeneity, evidenced by the shape of the tan δ curves and fwhm values, with similar damping characteristics as reflected in the peak of the tan δ peak.

### 3.5 Vitrimeric Behavior

Figure 5a shows the fitting of the stress relaxation results to the Arrhenius equation of each formulation, and Figure 5b presents the Angell fragility plot, showing the logarithm of viscosity as a function of *T*_g_·*T* ^*–1*^. No vitrimeric characterization was performed on DGEBA/IPDA because it lacks dynamic exchangeable groups in the network.

**Figure 5:**
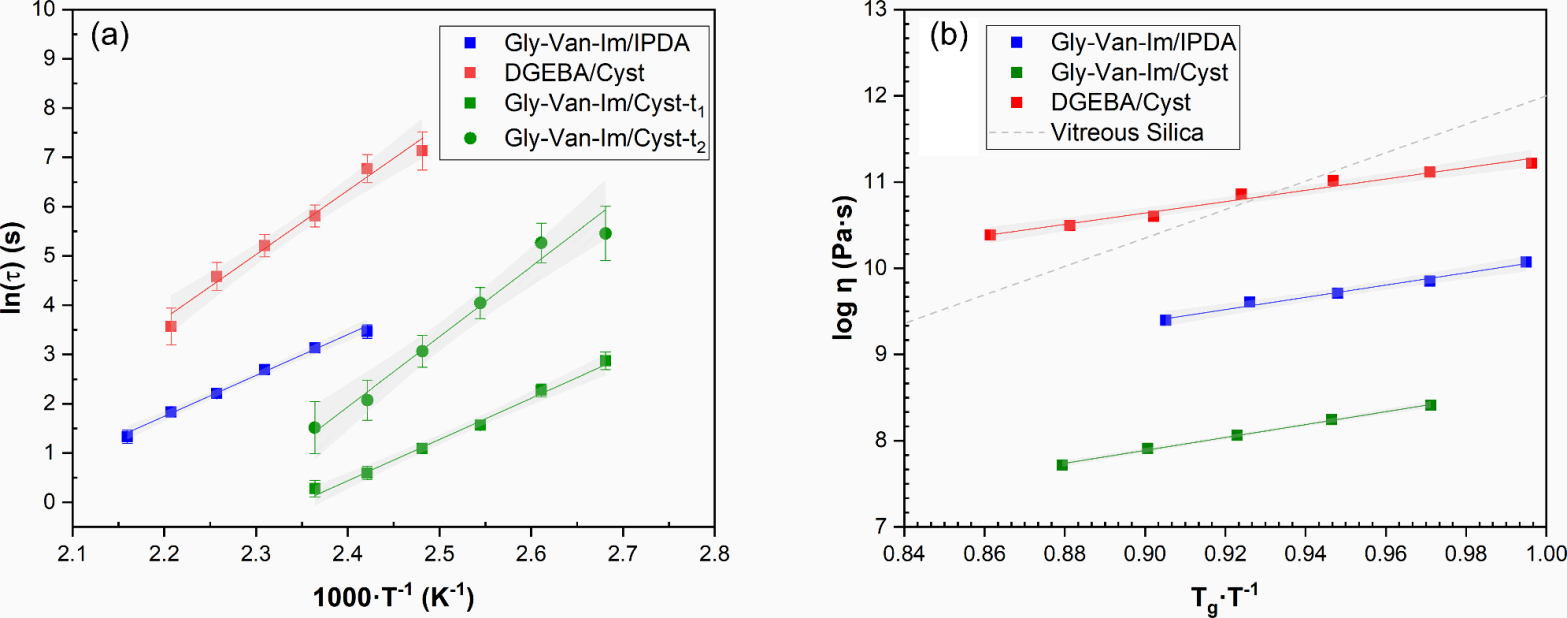
(a) Fitting of stress relaxation results to the Arrhenius equation for each formulation studied. (b) Angell fragility plot of the logarithm of the viscosity as a function of *T*_g_·*T* ^–1^. For comparative purposes, an ideal strong liquid is included as a reference (gray line).

The normalized stress relaxation profiles were first fitted to the KWW function (eq 1). Stress relaxation curves at various temperatures with the fitting by KWW functions are provided in Figures S23–S25 (Supporting Information) and the fitting parameters are listed in Table 4. The stretched parameter β shows values around 0.46 in the case of formulations Gly-Van-Im/IPDA and 0.66 in the case of DGEBA/Cyst. The *R* ^*2*^ showed values close to 1 in both cases, which indicates a good correlation between the model and the stress relaxation behavior. However, in the case of formulation Gly-Van-Im/Cyst, the values of *R* ^*2*^ indicated that the stretched exponential decay function (eq 1) did not precisely describe the relaxation process (results not shown). This different relaxation behavior is derived from the presence of two different exchange reaction mechanisms in formulation Gly-Van-Im/Cyst, imine and disulfide.

**Table 4:** Fitting Parameters for the Stretched Exponential KWW Decay Function, Activation Energies, and Topology Freezing Temperatures for Gly-Van-Im/IPDA and DGEBA/Cyst

Sample	*T* (°C)	β	τ (s)	*R* ^*2*^	ln *A*	*E*_*a*_ (kJ·mol^–1^)	*T*_*v*_ (°C)
Gly-Van-Im/IPDA	140	0.42	32	0.9982	16.4 ± 2.1	68.3 ± 0.9	35
	150	0.48	23	0.9947			
	160	0.49	15	0.9943			
	170	0.44	9	0.9900			
	180	0.40	6	0.9889			
	190	0.44	4	0.9936			
DGEBA/Cyst	120	0.74	1987	1.000	24.9 ± 5.8	108.0 ± 2.5	59
	130	0.68	1250	0.9997			
	140	0.61	870	0.9975			
	150	0.66	334	0.9989			
	160	0.66	183	0.9987			
	170	0.64	98	0.9968			
	180	0.67	35	0.9964			
	190	0.67	11	0.9901			

In this sense, a multimodal Maxwell model (eq 2) was used to fit to the stress relaxation curves of Gly-Van-Im/Cyst (Figure S24). The model is composed of two Maxwell components with two characteristic relaxation times, *τ*_*1*_ and *τ*_*2*_. The fitting parameters are presented in Table 5. The values of *R* ^*2*^ are close to 1 in each temperature which indicates a good correlation between the experimental behavior and the multimodal model.

**Table 5:** Fitted Parameters for the Multimodal Maxwell Equation, Activation Energies, and Topology Freezing Temperature

Formulation	*T* (°C)	*β*_*1*_	*τ*_*1*_ (s)	*β*_*2*_	*τ*_*2*_ (s)	*R* ^*2*^	ln *A*_*1*_	ln *A*_*2*_	*E*_*a1*_ (kJ·mol^–1^)	*E*_*a2*_ (kJ·mol^–1^)	*T*_*v*_ (°C)
Gly-Van-Im/Cyst	100	0.59	18	0.29	234	1.0000	19.6 ± 2.5	30.7 ± 7.7	69.4 ± 1.0	113.2 ± 3.1	–99
	110	0.69	10	0.41	193	0.9966					
	120	0.71	5	0.39	57	0.9987					
	130	0.71	3	0.38	21	0.9990					
	140	0.75	2	0.38	8	0.9996					
	150	0.77	1	0.39	5	0.9999					

The values of *E*_*a*_ obtained with the multimodal Maxwell model coincide with the values of *E*_*a*_ obtained with the KWW model for each exchange reaction mechanism: Gly-Van-Im/IPDA (imine exchange) showed *E*_*a*_ = 68.3 kJ·mol^–1^ and the first component of the mutilmodal model showed *E*_*a*_ = 69.4 kJ·mol^–1^; and DGEBA/Cyst (disulfide exchange) showed *E*_*a*_ = 108.0 kJ·mol^–1^ and the second component of the multimodal model showed *E*_*a*_ = 113.2 kJ·mol^–1^. However, the values of *E*_*a*_ corresponding to disulfide exchange mechanism obtained in this work do not correspond to those reported in the literature. Generally, the *E*_*a*_ of material with imine dynamic groups showed values around 70–80 kJ·mol^–1^,^49^ but the *E*_*a*_ of materials with disulfide dynamic bonds showed values of *E*_*a*_ = 55 kJ·mol^–1^.^50^ The difference may come from the number of dynamic groups present in the networks. Disulfide dynamic bonds only come from cystamine, which represents around a 17% of formulations Gly-Van-Im/Cyst and DGEBA/Cyst. Low disulfide content reduces the number of available exchange sites, which slows down network rearrangement and can increase the effective *E*_*a*_ for stress relaxation.^51,52^

Figures S26–S28 show the creep curves of the material studied. In the Angell plot (Figure 5b), Gly-Van-Im formulations display much lower viscosities than an ideal strong liquid, Gly-Van-Im/Cyst showing exceptionally low viscosities, highlighting the effect of dual dynamic groups.

Regarding the topology freezing temperature (*T*_v_), the values obtained are in accordance with the *E*_a_, less energy is needed for the bonds to exchange, then lower *T*_v_ is obtained. In the case of the Gly-Van-Im/Cyst formulation a *T*_v_ obtained was −99 °C, which is considerably lower than the Gly-Van-Im/IPDA and DGEBA/Cyst formulations (35 and 59 °C respectively). Such low *T*_v_ could be explained by the combination of two relaxation mechanism in the same network that impart a synergistic effect in the relaxation of the material.

### 3.6 Mechanical Recycling

The mechanical recyclability of the material was assessed using Gly-Van-Im/IPDA and Gly-Van-Im/Cyst materials. Samples were cut into small pieces, placed in a stainless-steel mold, and hot-pressed to obtain a circular sample of the recycled material (Figure S29).

The material was hot-pressed at 180 °C under 50 MPa for 1 h. The pressing temperature was selected to be above the *T*_g_, and the processing time was based on the stress relaxation profile. The applied pressure was arbitrary, to ensure proper contact between the material pieces. After pressing, the sample was cooled to room temperature and removed from the mold. The circular sample obtained was cut into a rectangular specimen for determining the thermomechanical properties by DMA. In Figure 6 it can be observed the storage modulus *E*′ and the damping factor (tan δ) of the recycled materials, and the main thermomechanical data obtained are listed in Table 6. A decrease in the tan δ peak can be observed upon recycling, from 128 to 120 °C for Gly-Van-Im, and from 98 to 88 °C for Gly-Van-Im/Cyst. In addition, the height of the tan δ peaks decrease while the curves broadened. Interestingly, there is a significant increase in the glassy modulus *E*′_*g*_, particularly for Gly-Van-Im/Cyst. This enhancement is likely due to the compression applied during recycling. Thanks to topological network rearrangement driven by imine-exchange reactions during compression at high temperature, the stacking and the density of the network increase, thereby increasing the rigidity at low temperatures.

**Figure 6:**
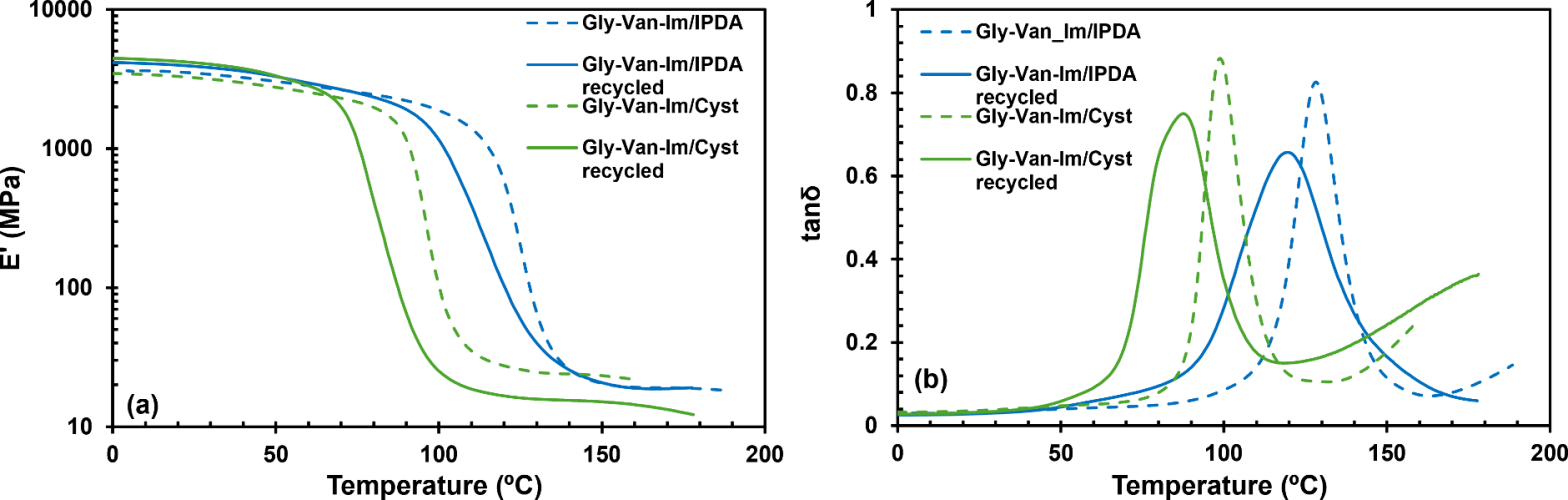
(a) Storage modulus E′ (a) and tan δ (b) as a function of temperature for the pristine and recycled materials.

**Table 6:** Thermomechanical Data of the Pristine and of the Recycled Thermosetting Polymers

Material	*T*_*tanδ*_ (°C)	fwhm (°C)	*E*′_*g*_ (MPa)	*E*′_*r*_ (MPa)
Gly-Van-Im/IPDA	128	17	2492	19
Gly-Van-Im/IPDA recycled	120	35	2674	19
Gly-Van-Im/Cyst	99	13	2778	23
Gly-Van-Im/Cyst recycled	88	25	3821	16

These observations suggest that some mechanical degradation occurred due to excessive shear during the cutting of the samples. However, this does not significantly affect the material properties, as the recycling process can still be optimized. In any case, the materials prepared with Gly-Van-Im demonstrated that reprocessing is feasible.

### 3.7 Chemical Degradation

A cured Gly-Van-Im/IPDA sample was used to test acid hydrolysis as a proof of concept for chemical degradability. This material was selected to avoid interference from disulfide groups, leaving the imine bond as the only labile group. The sample was immersed in 30 mL of 0.2 M HCl solution in a H_2_O:THF (2:8) mixture, and stirred at room temperature, following a procedure previously reported by our group.^8^ Figure 7 shows pictures of the sample at time 0 (Figure 7-a), and after 3 days at room temperature (Figure 7-b). After this period, only partial degradation was observed, as the solution turned colored, but the specimen remained practically intact. In our previous research of a monomer containing imine bonds, these conditions were enough for hydrolysis. We attribute these differences to a more compact structure that limits swelling using a H_2_O:THF (2:8) mixture, preventing the acidic water from reaching the imine bonds. To address this, the temperature was increased to 50 °C and, after 5 h at this temperature (Figure 7-c), the sample was completely degraded. However, the degradation products were not soluble in the degradation media and remained as a viscous precipitate at the bottom of the vial. The expected degradation products will be the corresponding aldehyde and ammonium salt derived from the hydrolysis of the imine,^53^ however, due to the high molecular weight of the degraded fragments (four fragments for each curing agent molecule) they were insoluble in the degradation media. This insolubility is not necessarily a drawback. Nevertheless, this test demonstrates that the material can be hydrolyzed under mild conditions, which could be advantageous for recovering fillers if the material is used as a matrix in composites.

**Figure 7:**
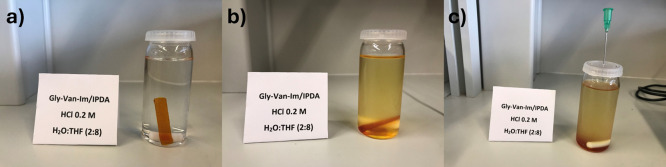
Cured sample introduced into 30 mL of a 0.2 M HCl solution in a H_2_O:THF (2:8) mixture. (a) time 0, (b) 3 days at room temperature, and (c) 5 h at 50 °C.

## 4 Conclusions

We have reported the preparation of an epoxy monomer derived exclusively from renewable feedstocks (biobased vanillin and epichlorohydrin). The synthetic route was designed with sustainability in mind, employing renewable solvents (water, ethanol, or solvent-free conditions) and catalytic hydrogenation (H_2_ with Pd/C) to maximize atom economy. The overall yield is reasonable (25%), for a four-step process, though the final recrystallization step accounts for significant losses, leaving room for optimization. The monomer synthesized was formulated with both IPDA, as a common amine hardener, and cystamine, as a biobased hardener. The resulting thermomechanical properties were benchmarked against those of DGEBA, the petrol-based epoxy monomer. Although DGEBA-based materials present a higher *T*_g_, the *T*_g_ values of the biobased–based materials, particularly with cystamine, were comparable, indicating strong potential for substitution in selected applications. Furthermore, the incorporation of imine bonds in the monomer structure imparts excellent vitrimeric behavior, with short relaxation times at relatively low temperatures. This enables both mechanical recycling and chemical degradation under mild conditions. Taken together, these results highlight a fully biobased epoxy system (in the case of cystamine formulations) that combines competitive thermomechanical performance with reusability and chemical removability, making it a promising candidate for applications such as reversible adhesives and recyclable composite matrices.

## Supplementary Material



## Notes

Figure S1, ^1^H NMR spectrum of Van-Ox in DMSO-*d*_6_; Figure S2, ^13^C NMR spectrum of Van-Ox in DMSO*-d*_6_; Figure S3, DSC thermogram of Van-Ox showing the melting point endotherm; Figure S4, ESI-MS spectrum of Van-Ox [M+H^+^]; Figure S5, ^1^H NMR spectrum of Van-NH_3_^+^Cl^–^ in DMSO-*d*_6_; Figure S6, ^13^C NMR spectrum of Van-NH_3_^+^Cl^–^ in DMSO*-d*_6_; Figure S7, ESI-MS spectrum of Van-NH_3_^+^Cl^–^ [M+H^+^]; Figure S8, ^1^H NMR spectrum of Van-Im in DMSO-*d*_6_ *;* Figure S9, ^13^C NMR spectrum of Van-Im in DMSO-*d*_6_; Figure S10, COSY spectrum of Van-Im in DMSO-*d*_6_; Figure S11, HSQC spectrum of Van-Im in DMSO-*d*_6_; Figure S12, HMBC spectrum of Van-Im in DMSO-*d*_6_; Figure S13, DSC thermogram of Van-Im showing the melting point endotherm; Figure S14, ESI-MS spectrum of Van-Im [M+H^+^]; Figure S15, COSY spectrum of pure Gly-Van-Im in CDCl_3_; Figure S16, HSQC spectrum of pure Gly-Van-Im in CDCl_3_; Figure S17, HMBC spectrum of pure Gly-Van-Im in CDCl_3_; Figure S18, DSC thermogram of pure Gly-Van-Im showing the melting point endotherm; Figure S19, ESI-MS spectrum of pure Gly-Van-Im [M+H^+^]; Figure S20, ^1^H NMR spectrum in DMSO-*d*_6_ of the Gly-Van-Im crude product after solvent evaporation; Figure S21, superposed DSC thermograms of cured samples of the prepared formulations indicating the *T*_g_; Figure S22, TGA isotherms of the prepared formulations at 180 °C for 3 h; Figure S23, stress relaxation curves at different temperatures of formulation Gly-Van-Im/IPDA; Figure S24, stress relaxation curves at different temperatures of formulation Gly-Van-Im/Cyst; Figure S25, stress relaxation curves at different temperatures of formulation DGEBA/Cyst; Figure S26, creep curves of formulation Gly-Van-Im/IPDA at temperatures from 120 to 160 °C; Figure S27, creep curves of formulation G*ly*-Van-Im/Cyst at temperatures from 110 to 150 °C; Figure S28, creep curves of formulation DGEBA/Cyst at temperatures from 120 to 190 °C; and Figure S29, Gly-Van-Im/Cyst material cut into small pieces and after mechanical recycling through hot-pressing (PDF)
