# Supplementary material for: Fully Biobased Epoxy–Imine Monomer: Synthesis and Curing with Amines Toward Vitrimeric Materials
Source: ACS Sustain Chem Eng. 2026 Feb 4;14(6):3222–34. doi: 10.1021/acssuschemeng.5c13119 (PMC12918244; doi:10.1021/acssuschemeng.5c13119)
Supplement: Supplementary file 1 [file sc5c13119_si_001.pdf]

Fully Biobased Epoxy–Imine Monomer: Synthesis and Curing with Amines Toward Vitrimeric Materials

Supporting information

Pere Verdugo[1,2,], Núria Montesó[1,2], Dailyn Guzman[1,2], David Santiago[1,3], Silvia De la Flor[3], Àngels Serra[2]*

[1] Eurecat, Technology Center of Catalonia - Chemical Technologies Unit, c/Marcel·lí Domingo 2, 43007 Tarragona, Spain.

[2] Universitat Rovira i Virgili, Department of Analytical and Organic Chemistry, c/Marcel·lí Domingo 1, 43007 Tarragona, Spain.

[3] Universitat Rovira i Virgili, Department of Mechanical Engineering, Av. Països Catalans 26, 43007 Tarragona, Spain.

*Corresponding author: pere.verdugo@eurecat.org

Phone number: +34 977 29 70 17 ext. 4536

Keywords: vanillin, epoxy, vitrimers, imine, sustainable, DGEBA, cystamine.

Number of pages: 16

Number of figures: 29

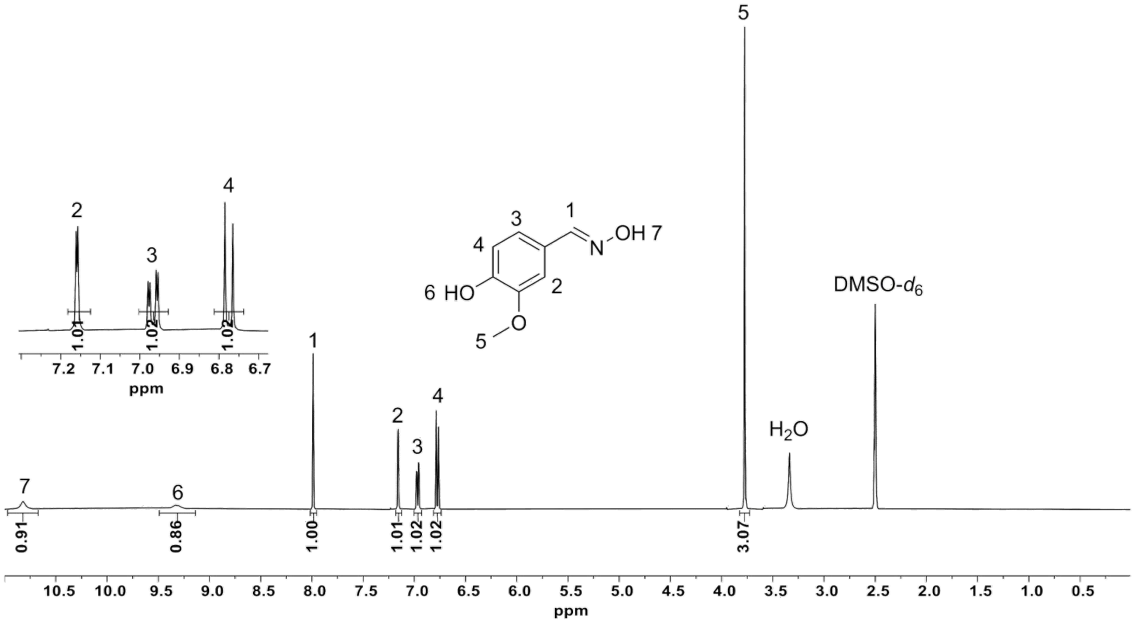

Figure S1. ^{1}H-NMR spectrum of Van-Ox in DMSO-d_6.

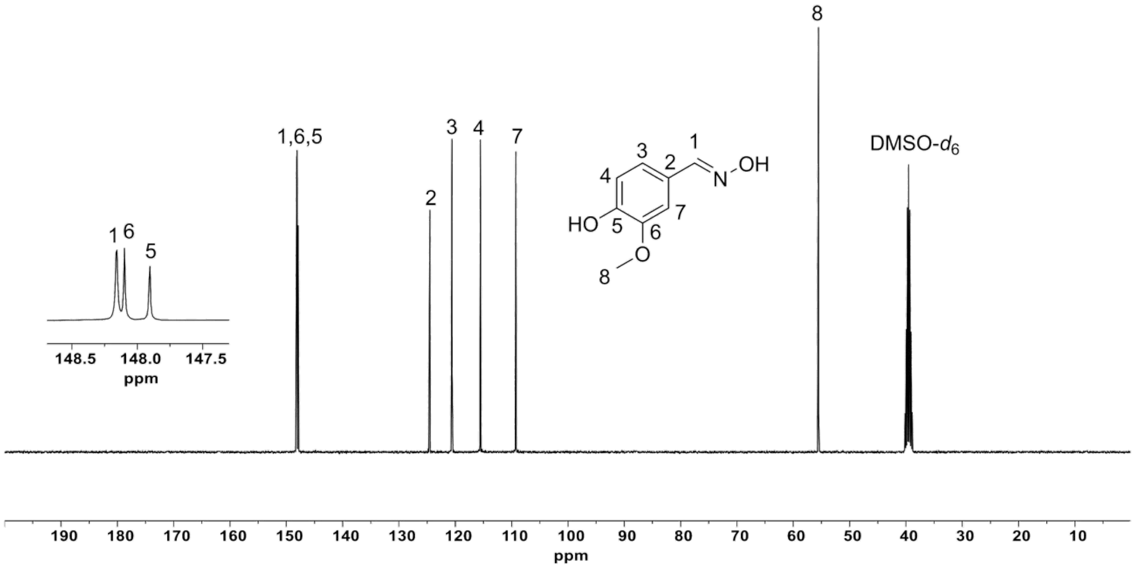

Figure S2. ^{13}C-NMR spectrum of Van-Ox in DMSO-d_6.

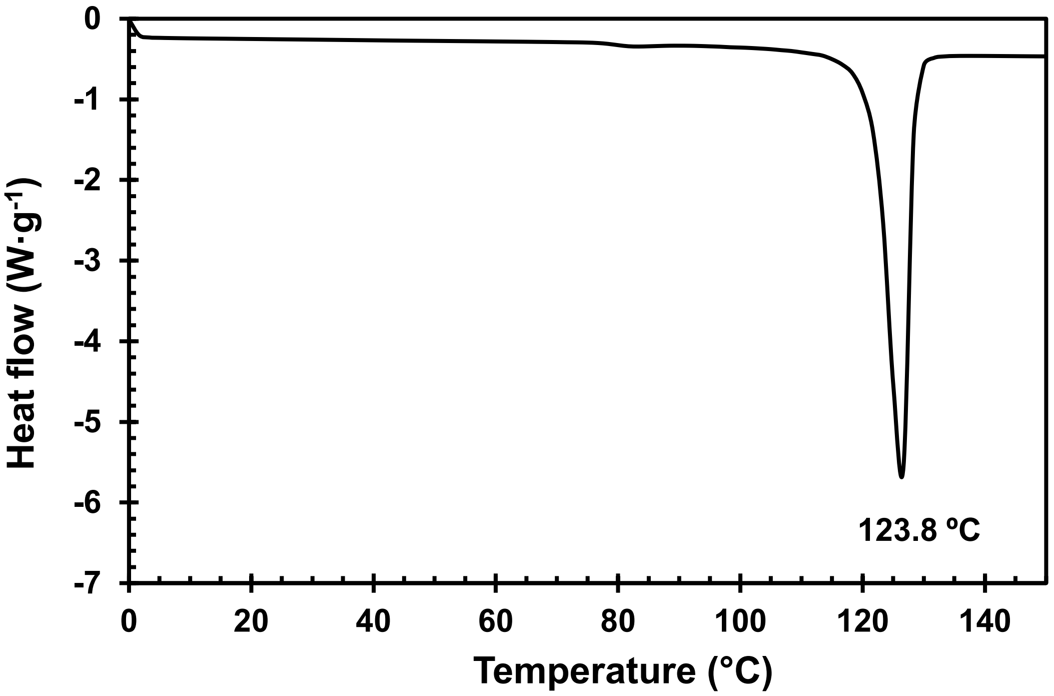

Figure S3 DSC thermogram of Van-Ox showing the melting point endotherm.

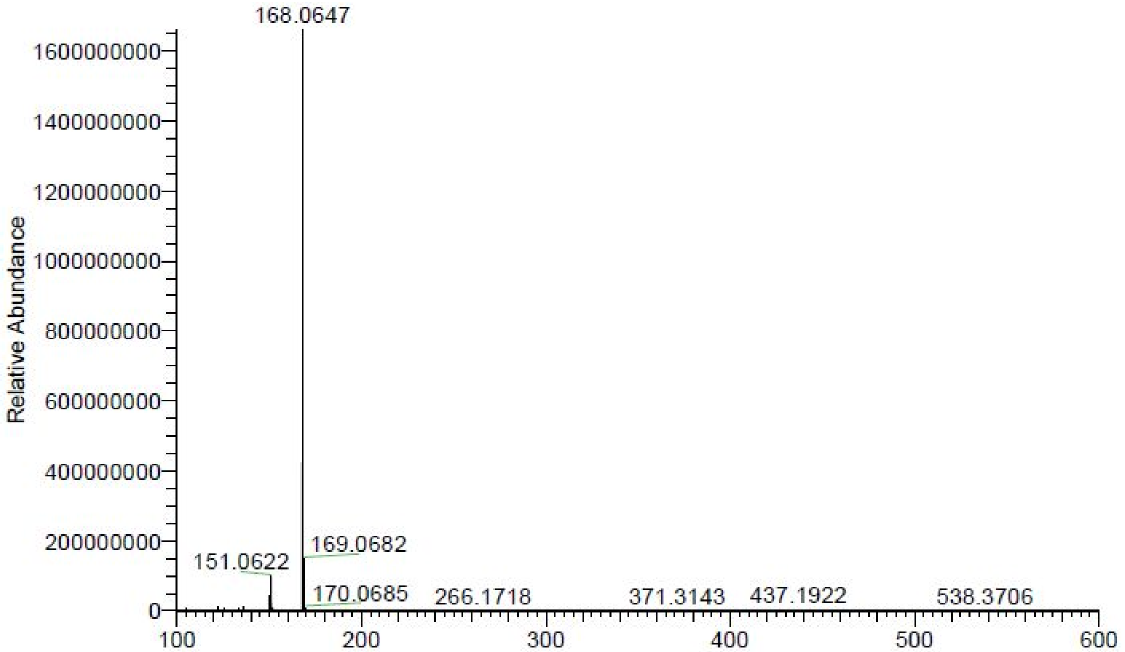

Figure S4. ESI-MS spectrum of Van-Ox [M+H+].

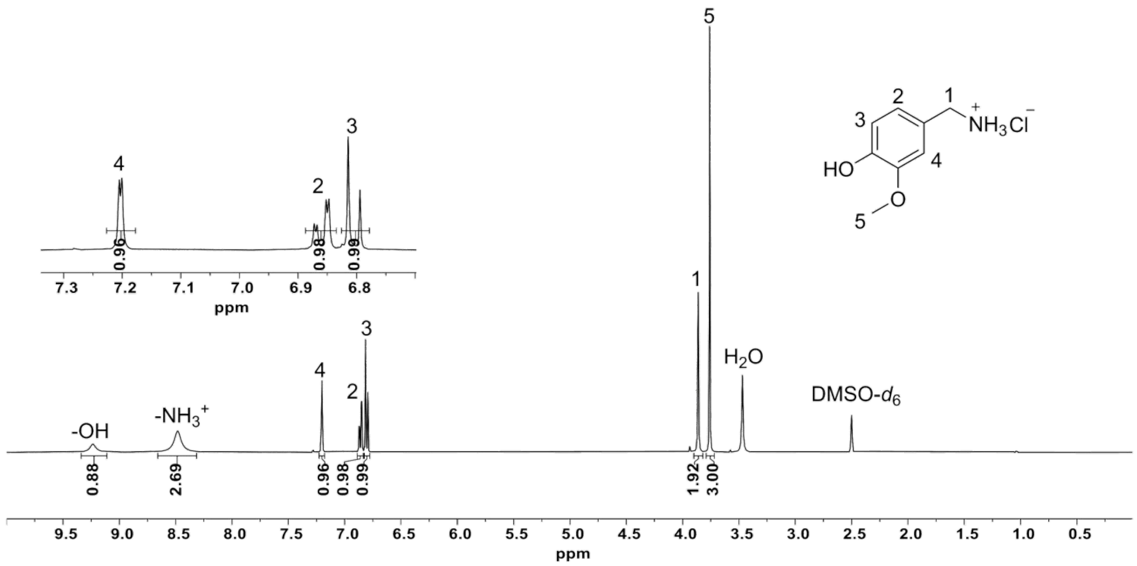

Figure S5. ^{1}H-NMR spectrum of Van-$NH_3^+Cl^-$ in DMSO-d_6.

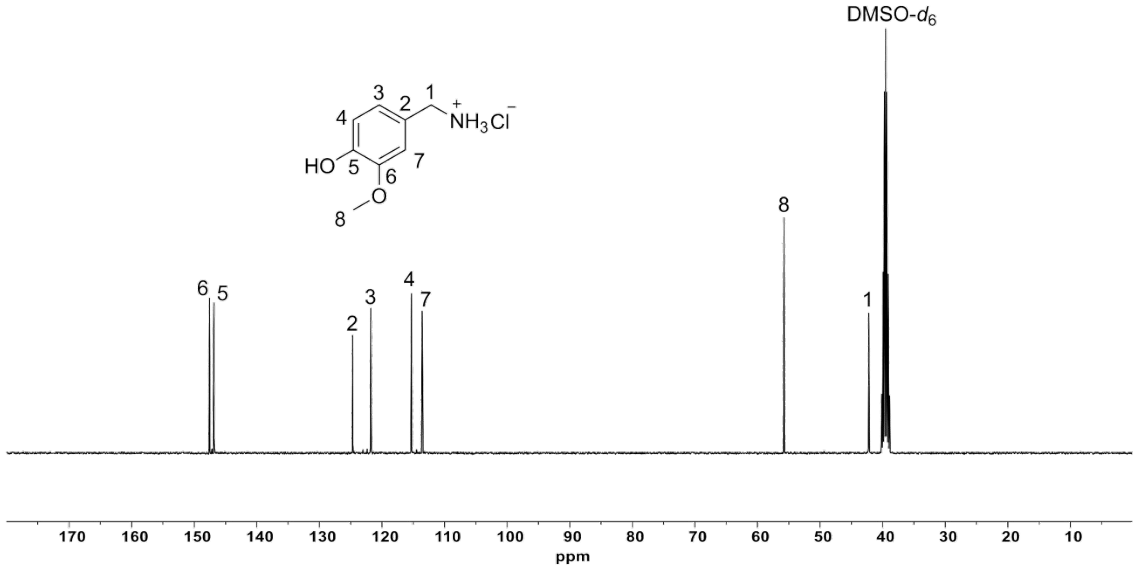

Figure S6. ^{13}C-NMR spectrum of Van-$NH_3^+Cl^-$ in DMSO-d_6.

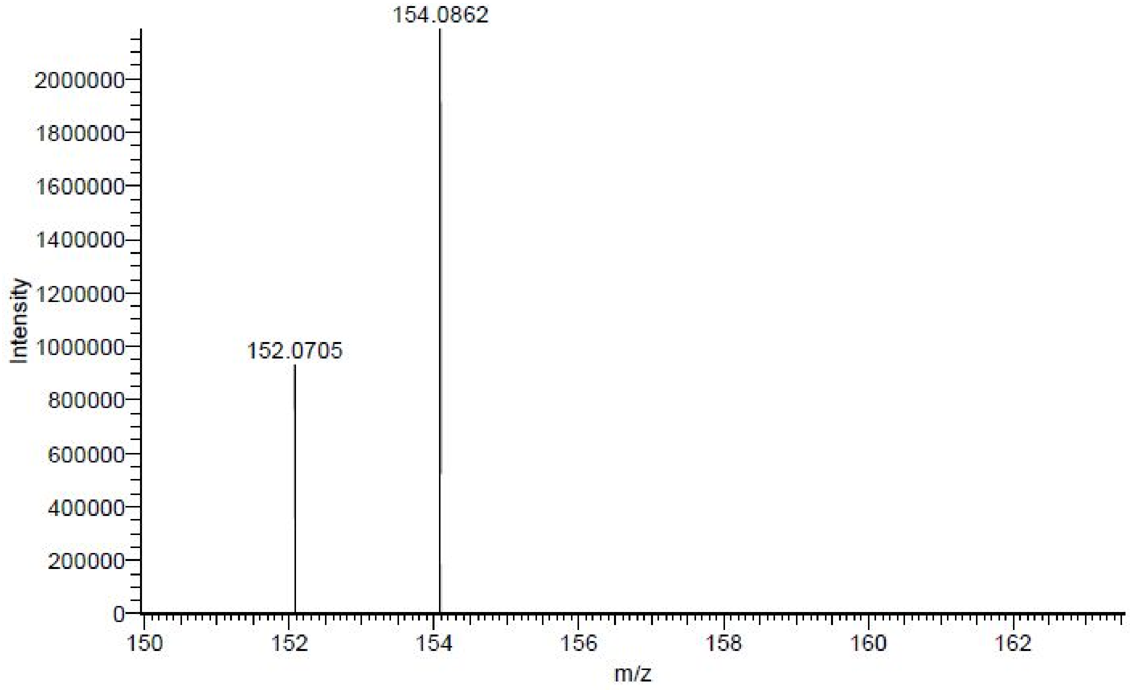

Figure S7. ESI-MS spectrum of Van-$NH_3^+Cl^-$ [M+H+].

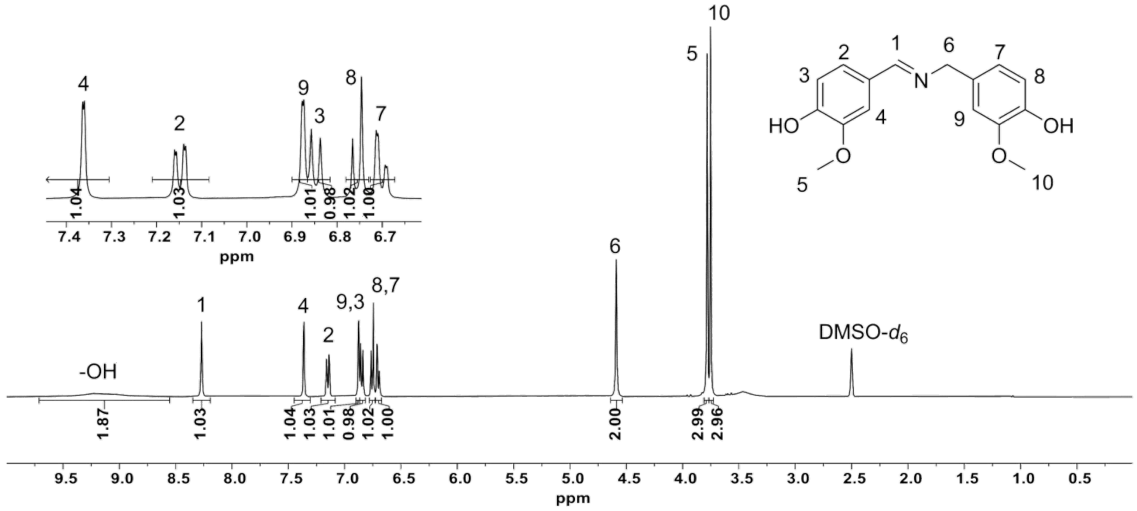

Figure S8 ^{1}H-NMR spectrum of Van-Im in DMSO-d_6.

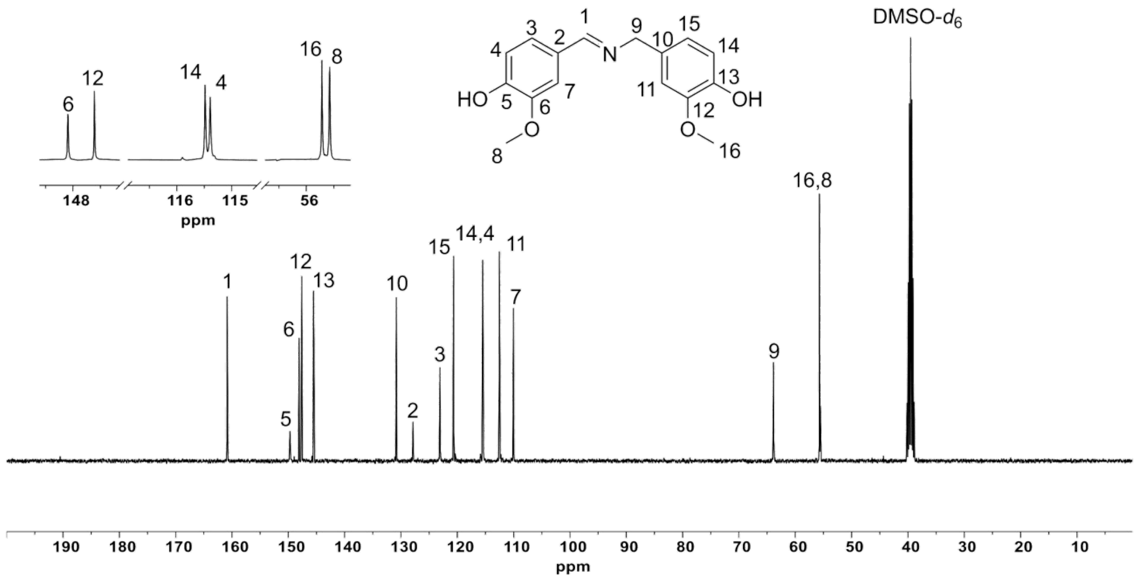

Figure S9 ^{13}C-NMR spectrum of Van-Im in DMSO-d_6.

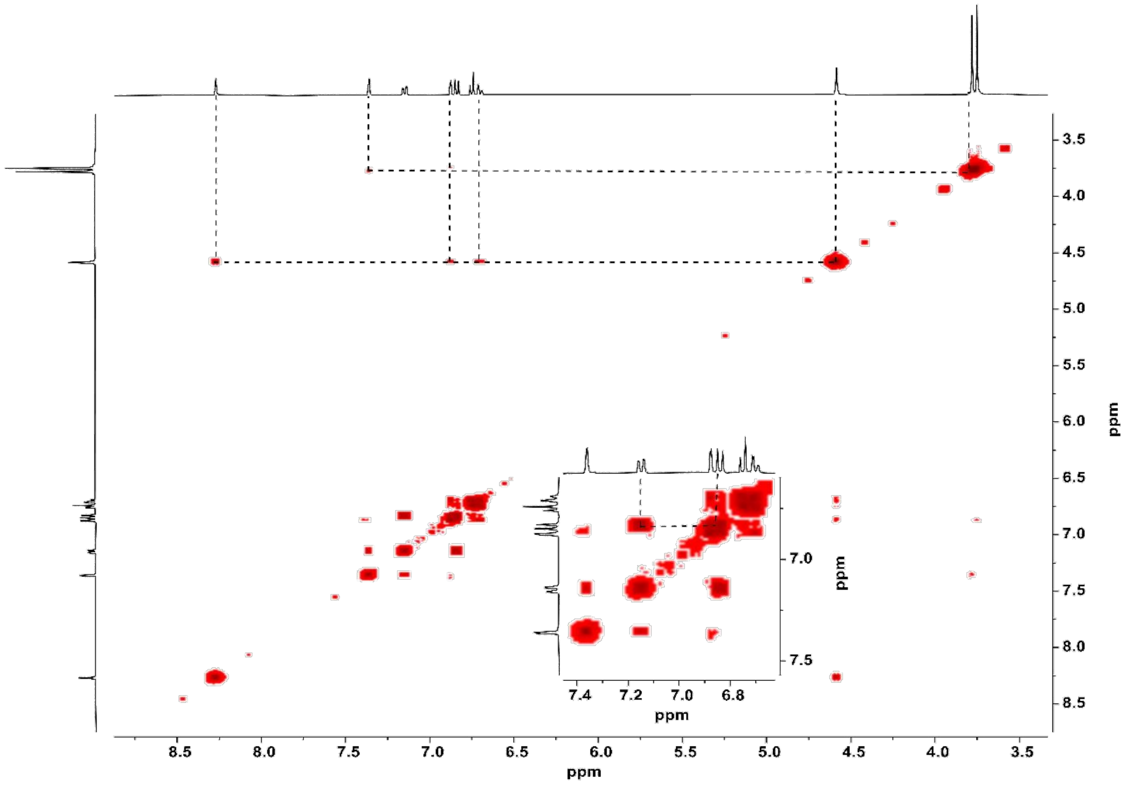

Figure S10 COSY spectrum of Van-Im in DMSO-d_6.

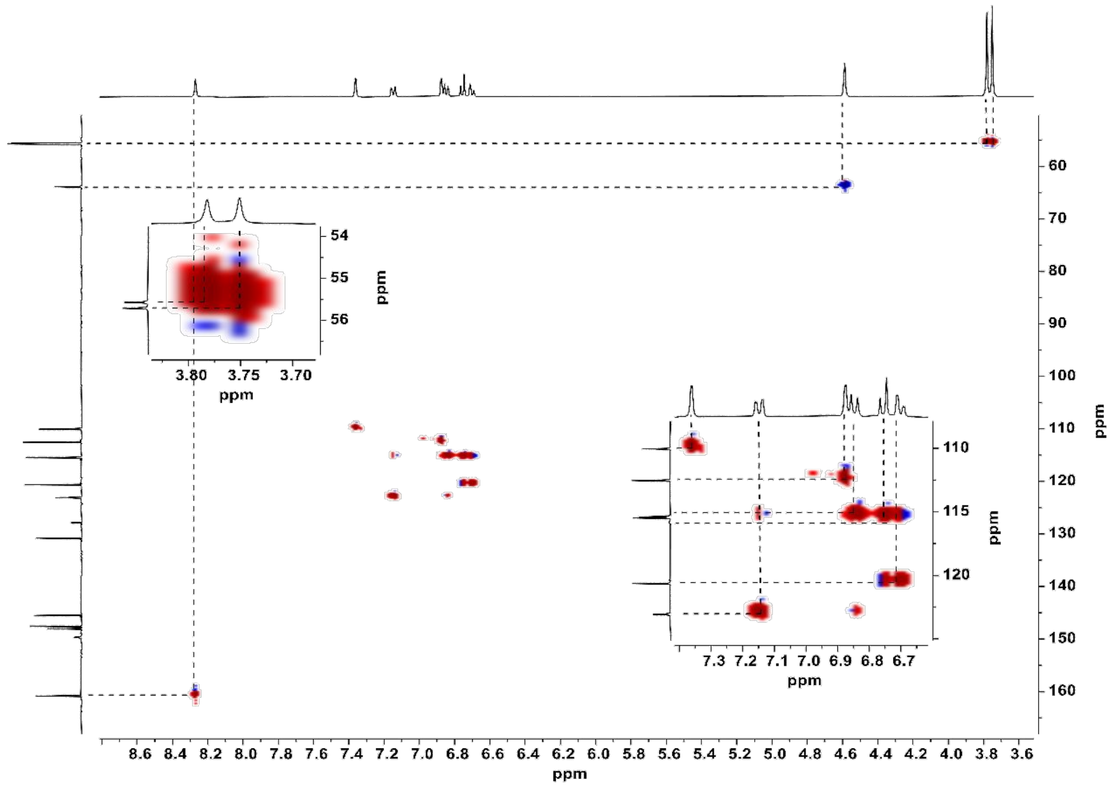

Figure S11 HSQC spectrum of Van-Im in DMSO-d_6.

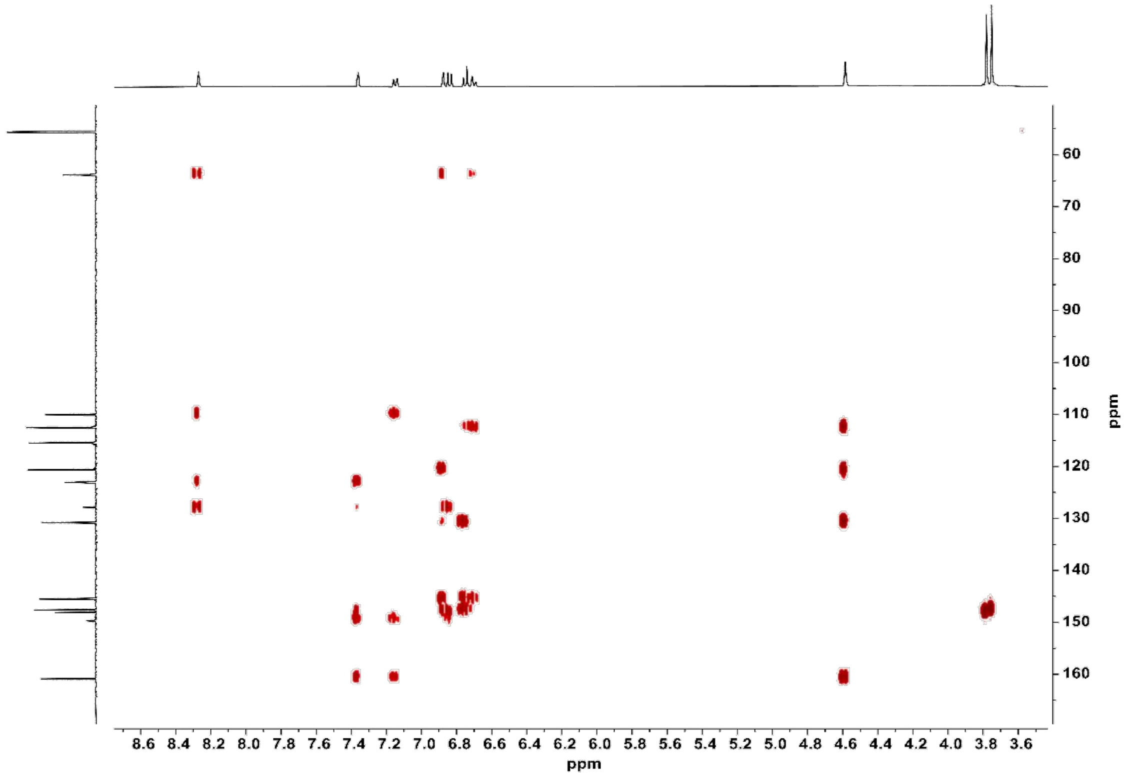

Figure S12 HMBC spectrum of Van-Im in DMSO-d_6.

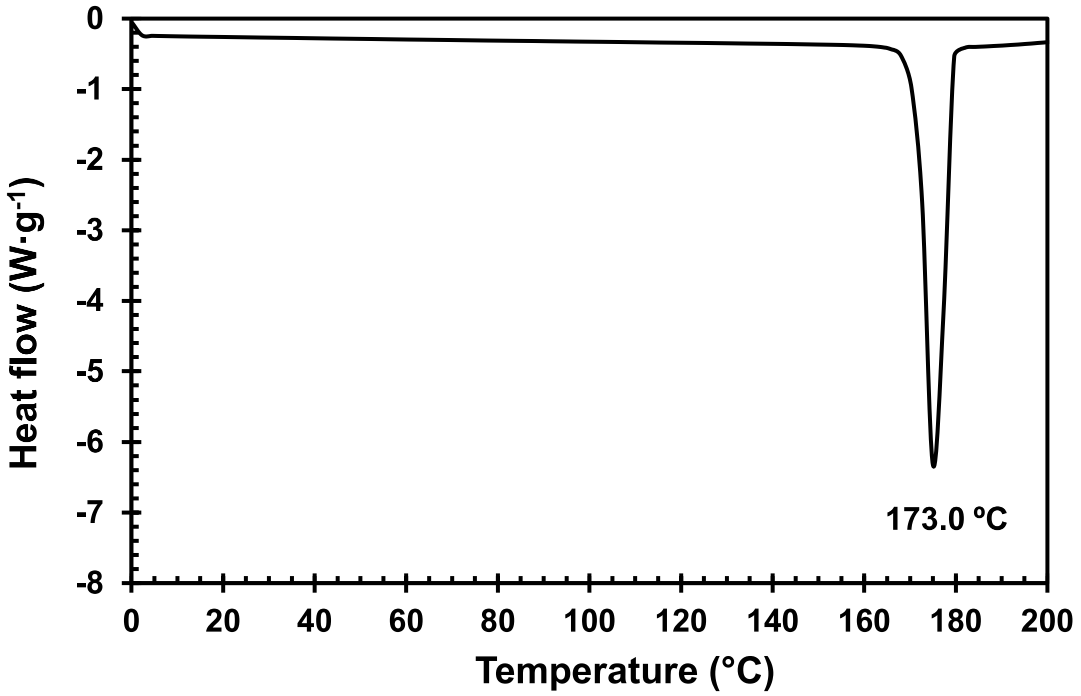

Figure S13 DSC thermogram of Van-Im showing the melting point endotherm.

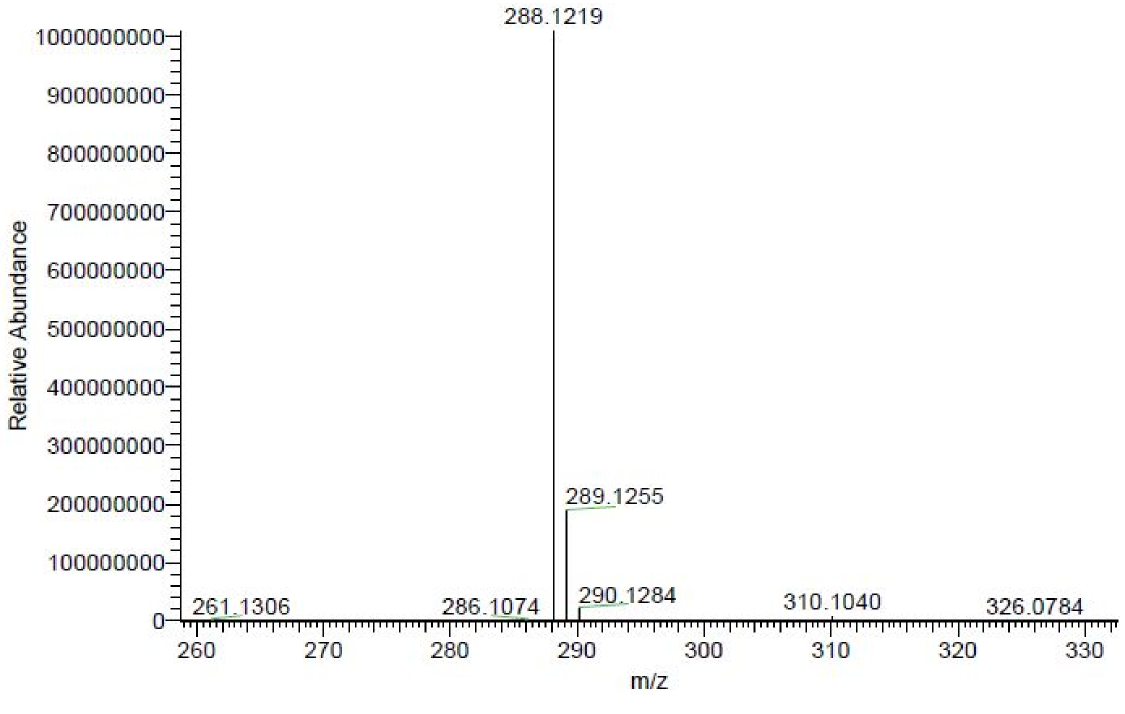

Figure S14 ESI-MS spectrum of Van-Im [M+H+].

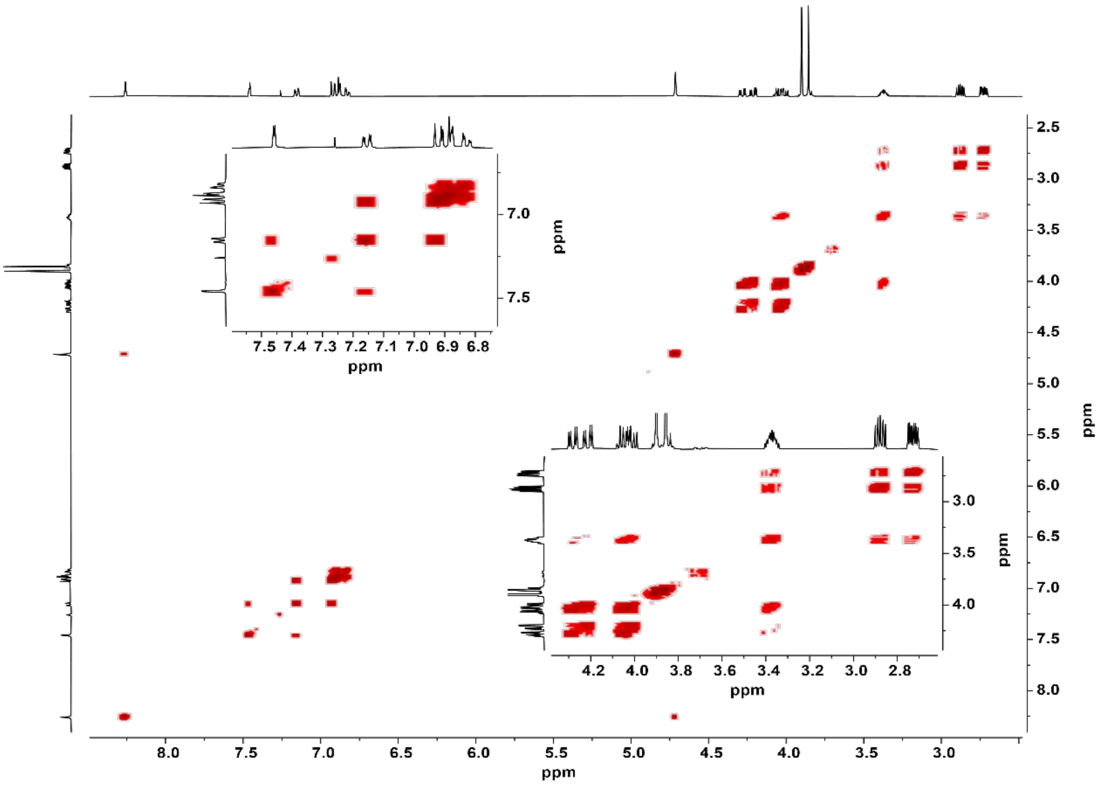

Figure S15 COSY spectrum of pure Gly-Van-Im in $CDCl_3$.

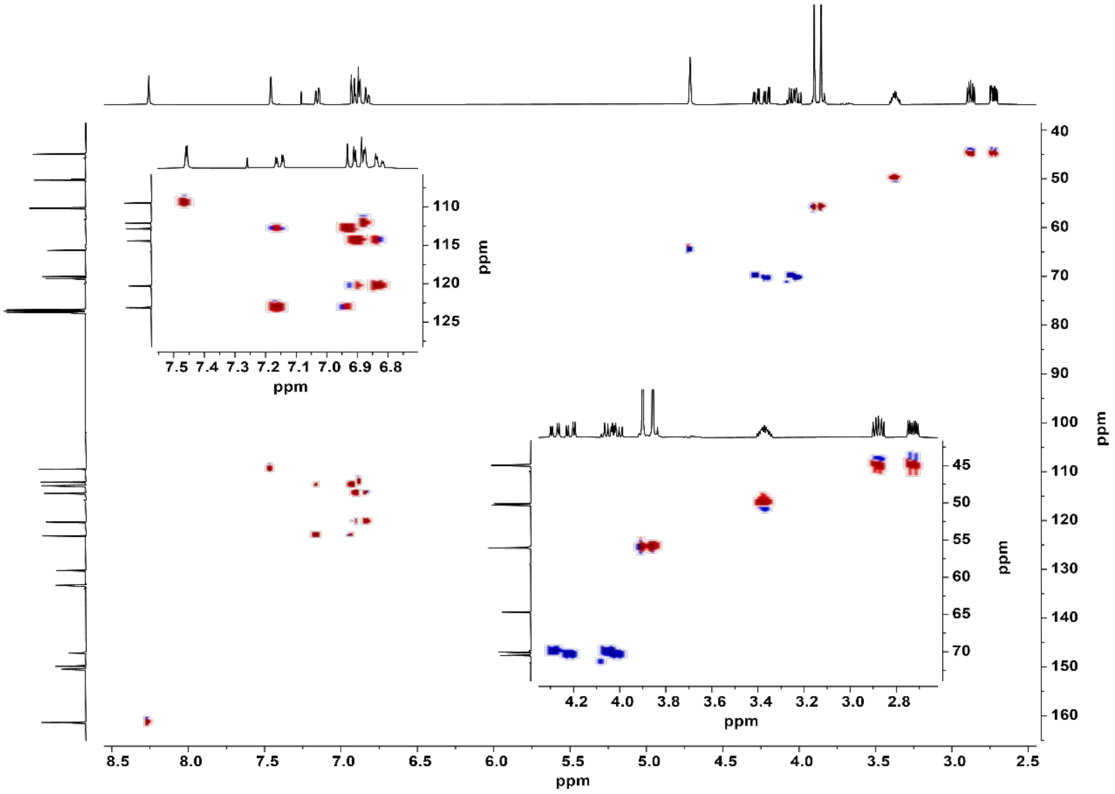

Figure S16 HSQC spectrum of pure Gly-Van-Im in $CDCl_3$.

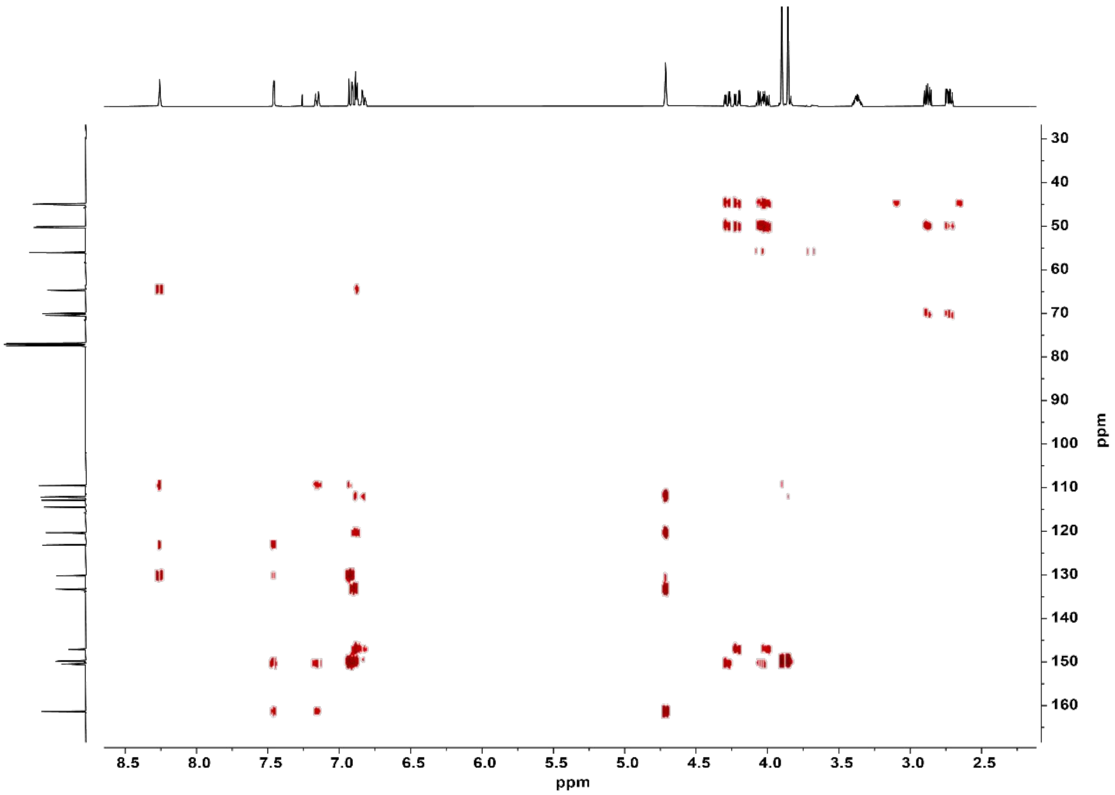

Figure S17 HMBC spectrum of pure Gly-Van-Im in $CDCl_3$.

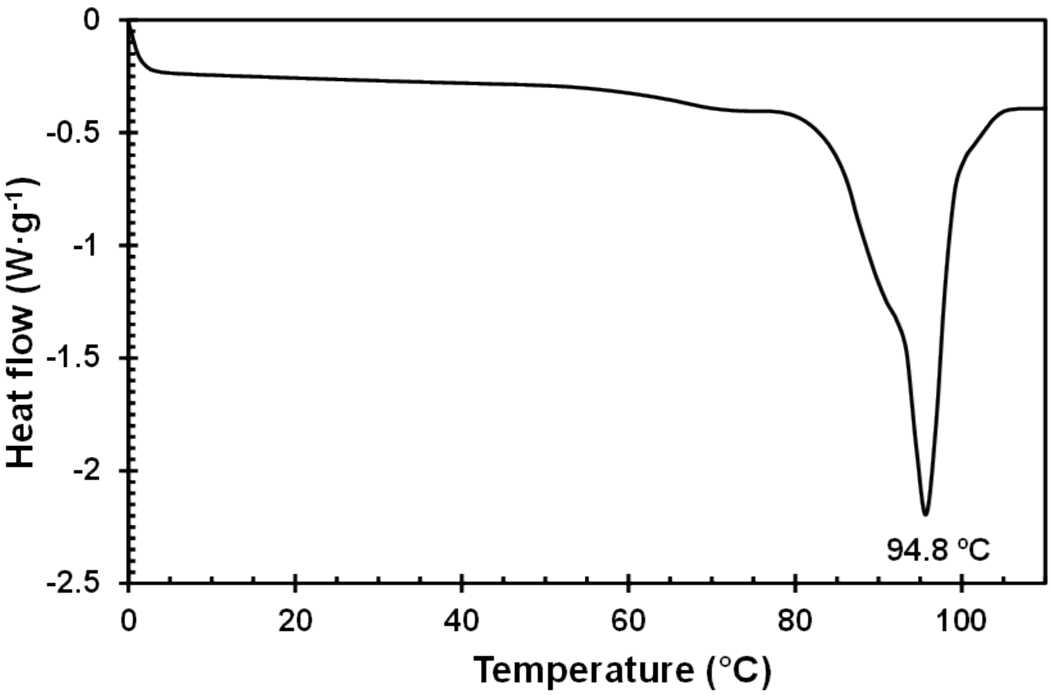

Figure S18 DSC thermogram of pure Gly-Van-Im showing the melting point endotherm.

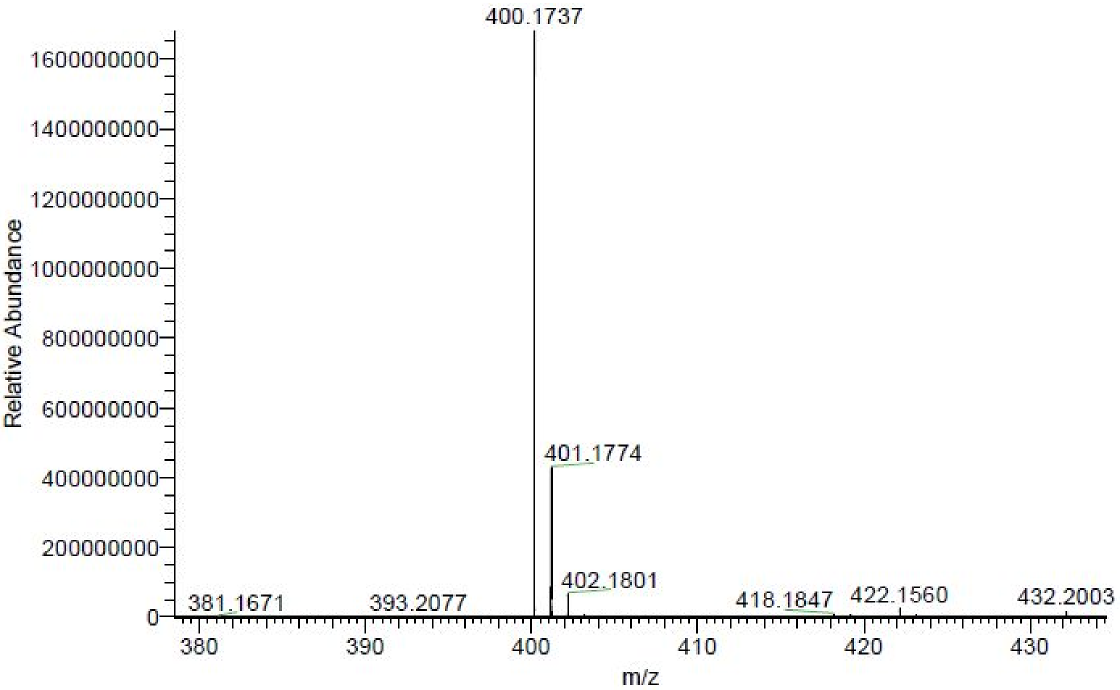

Figure S19 ESI-MS spectrum of pure Gly-Van-Im [M+H+].

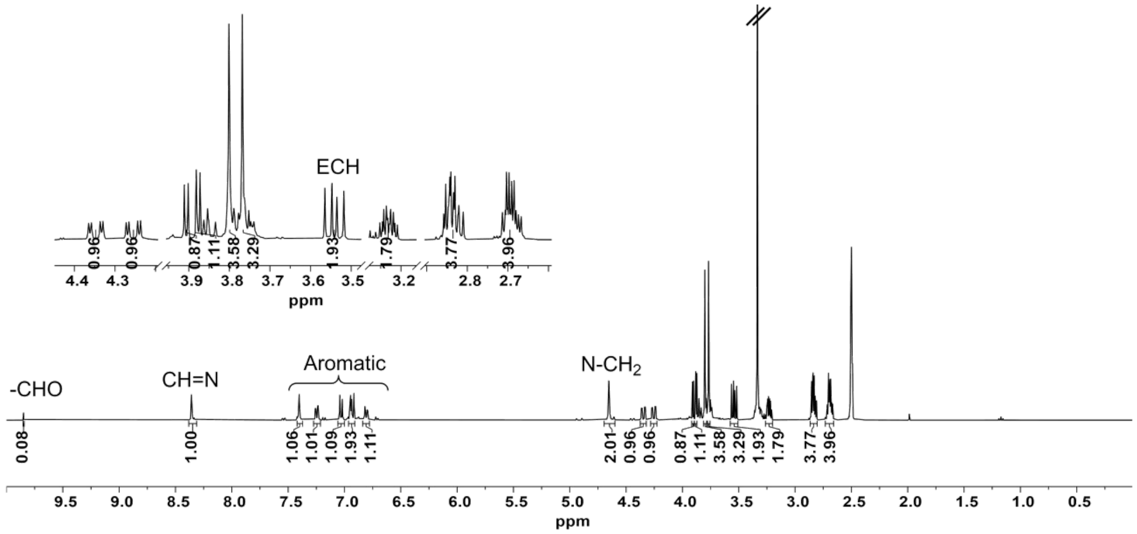

Figure S20 ^{1}H-NMR spectrum in DMSO-d_6 of the Gly-Van-Im crude product after solvent evaporation.

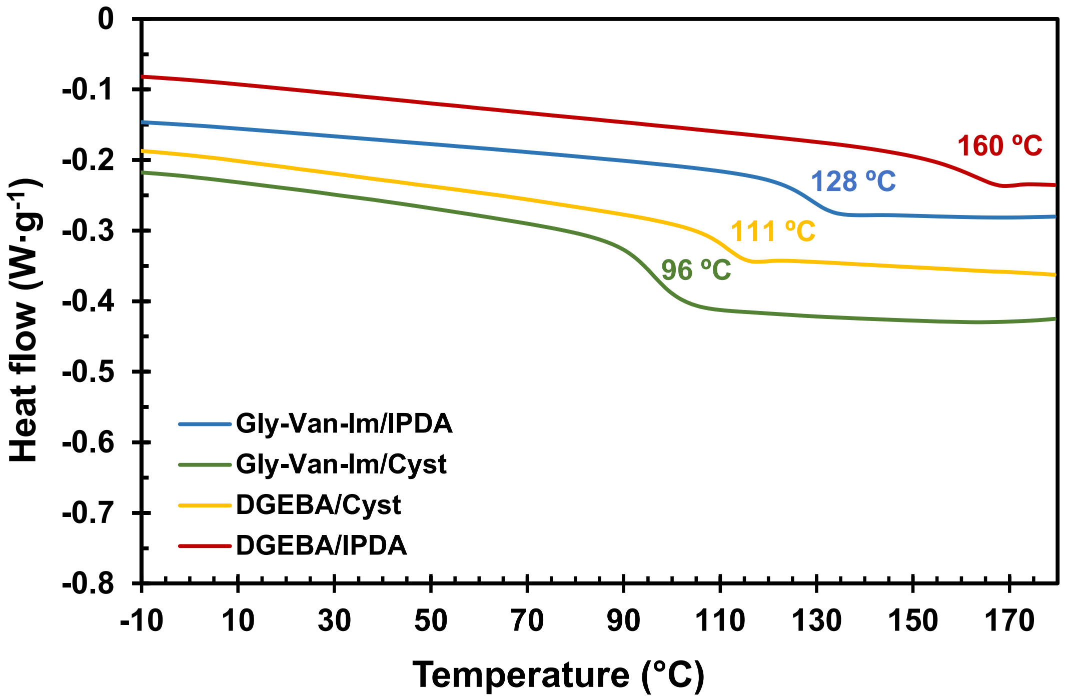

Figure S21 Superposed DSC thermograms of cured samples of the prepared formulations indicating the T_g.

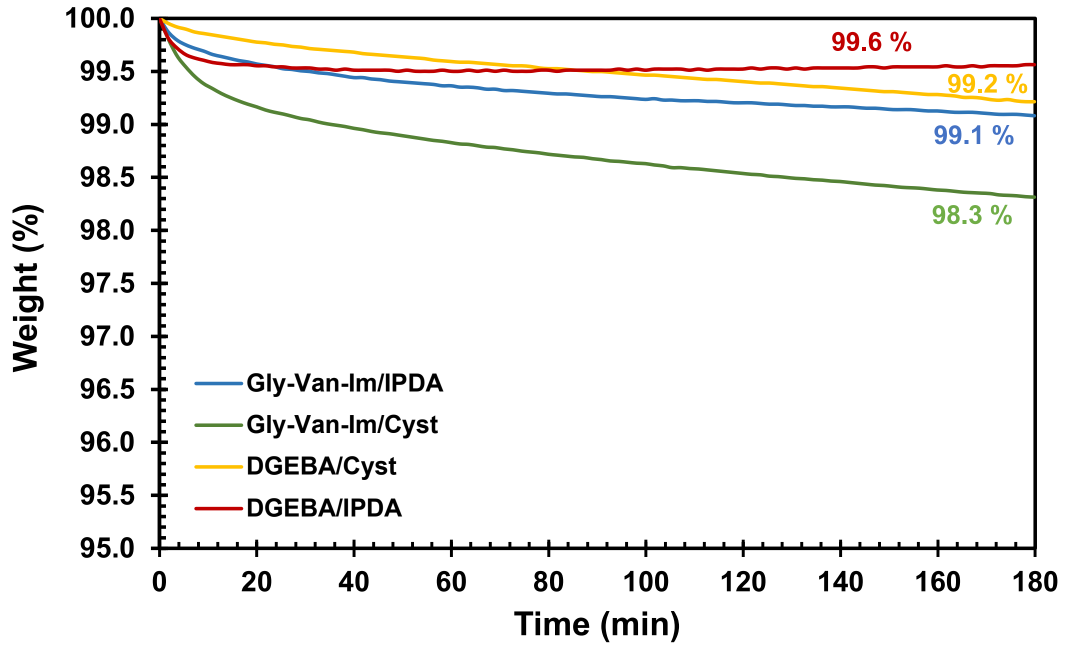

Figure S22 TGA isotherms of the prepared formulations at 180 °C for 3 hours.

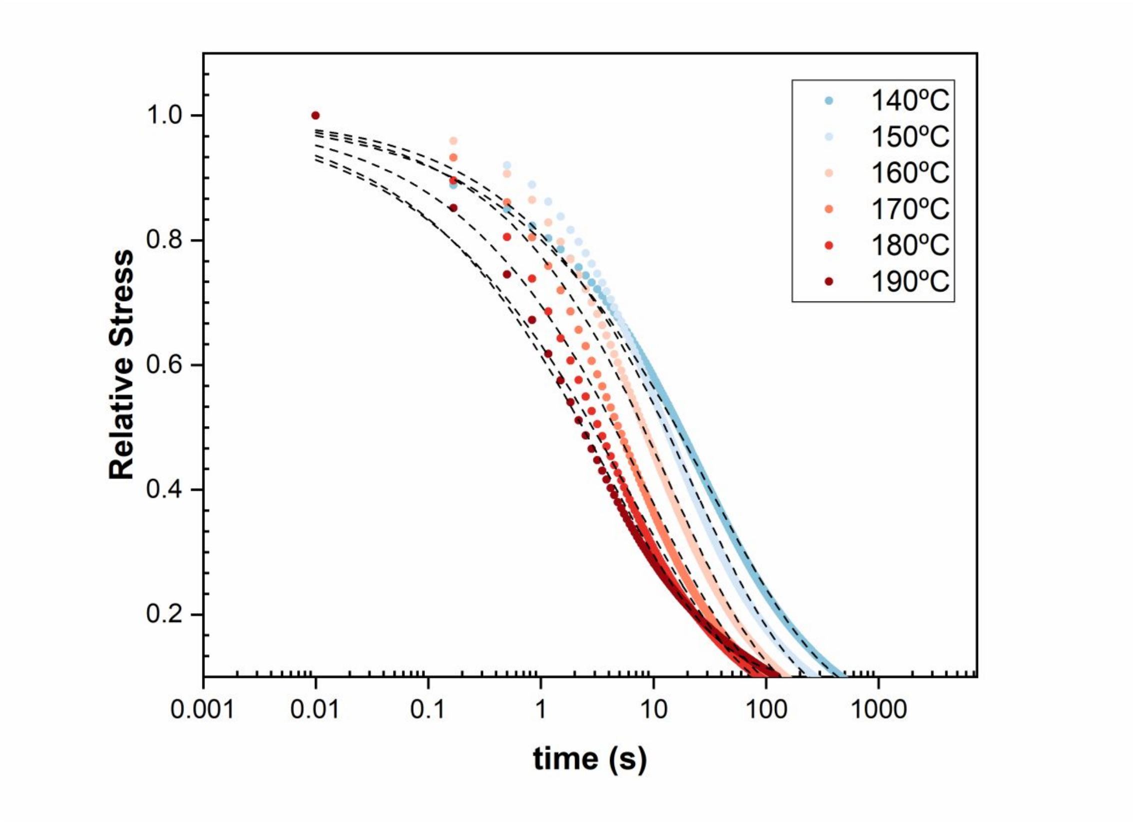

Figure S23 Stress relaxation curves at different temperatures of formulation Gly-Van-Im/IPDA.

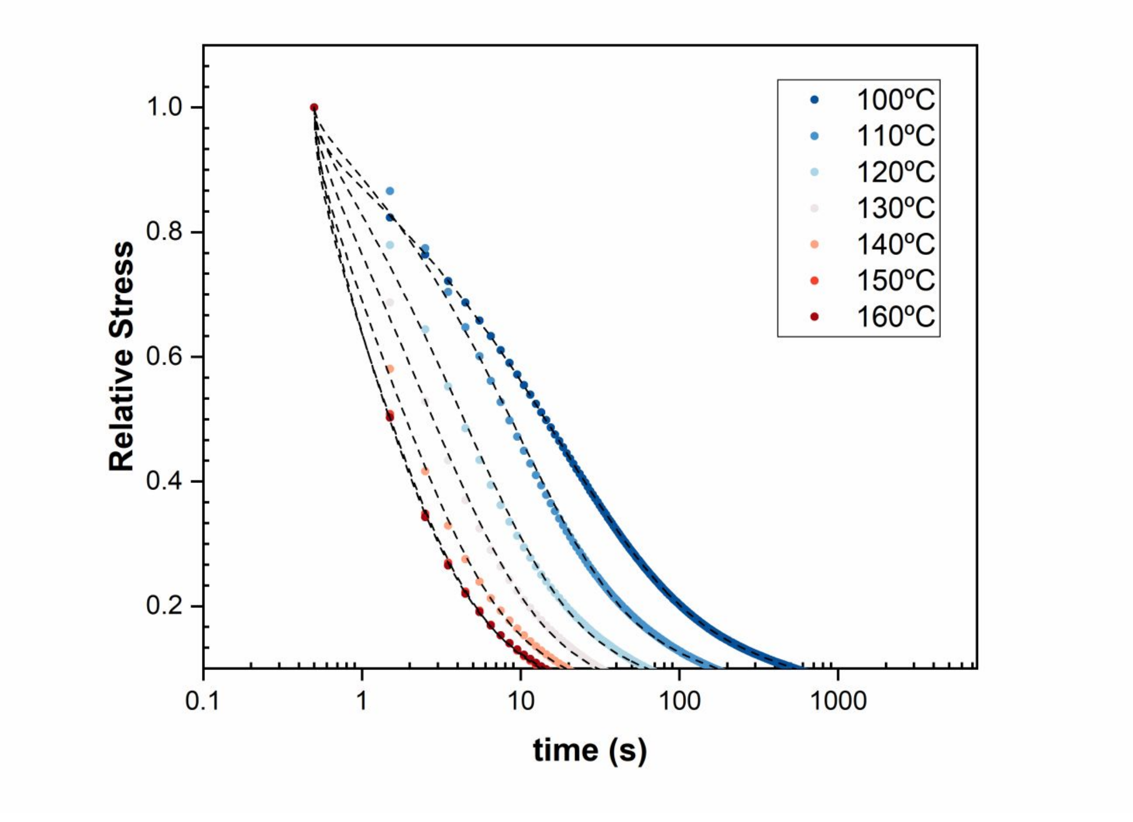

Figure S24 Stress relaxation curves at different temperatures of formulation Gly-Van-Im/Cyst.

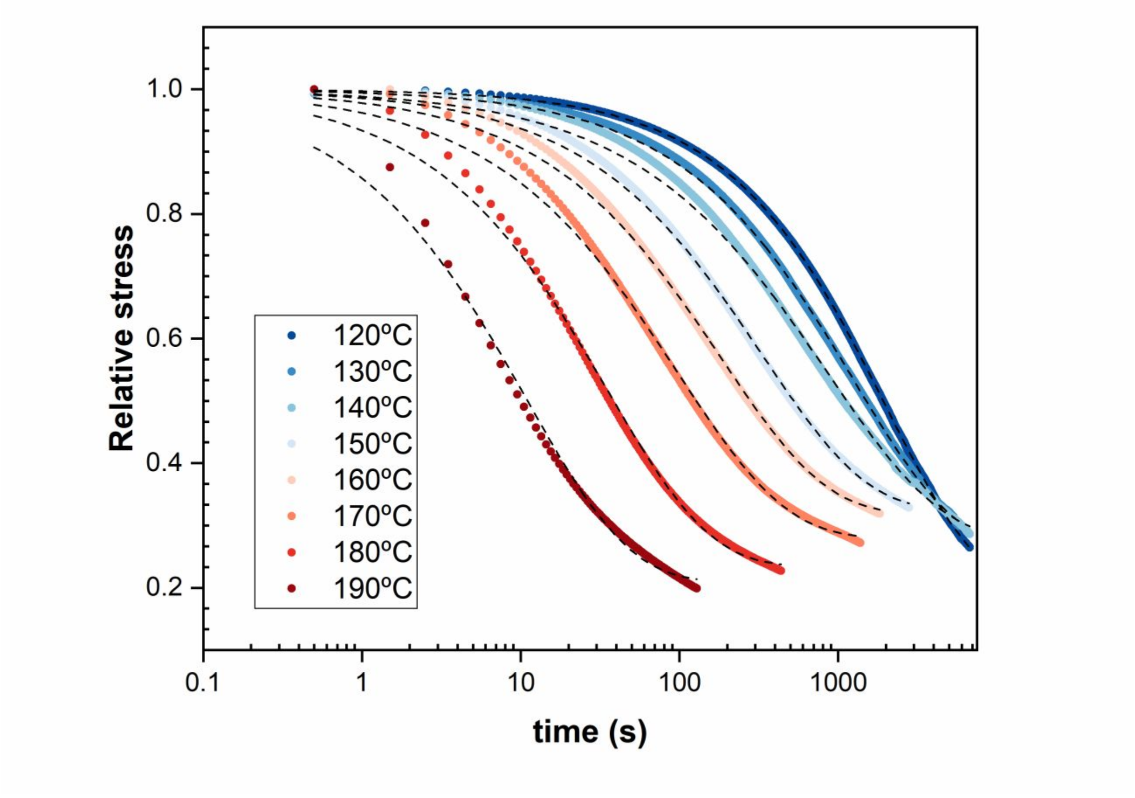

Figure S25 Stress relaxation curves at different temperatures of formulation DGEBA/Cyst.

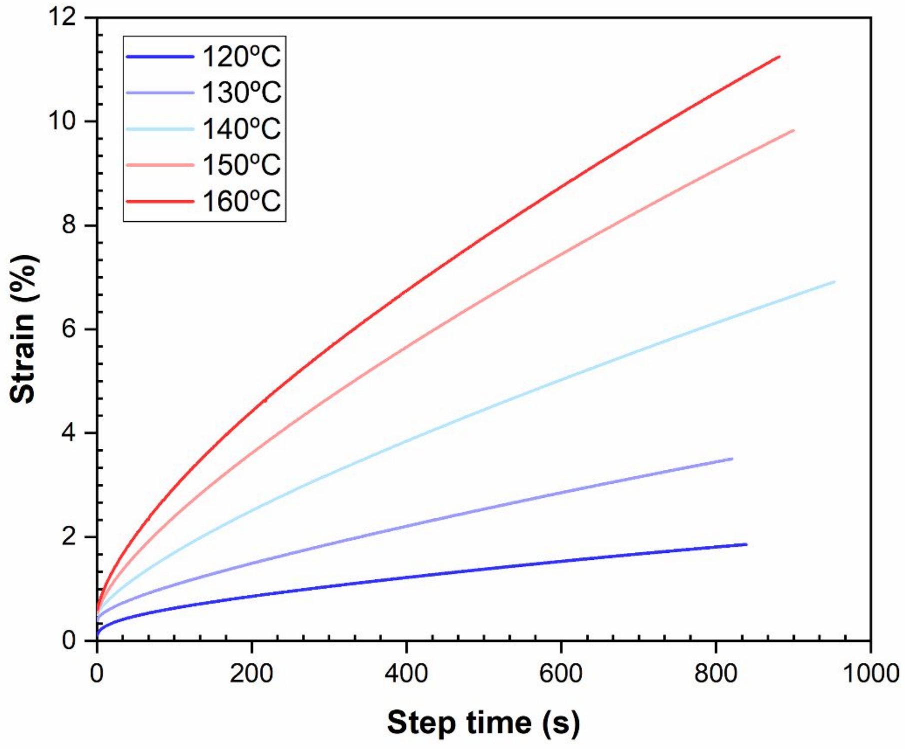

Figure S26 Creep curves of formulation Gly-Van-Im/IPDA at temperatures from 120 °C to 160 °C.

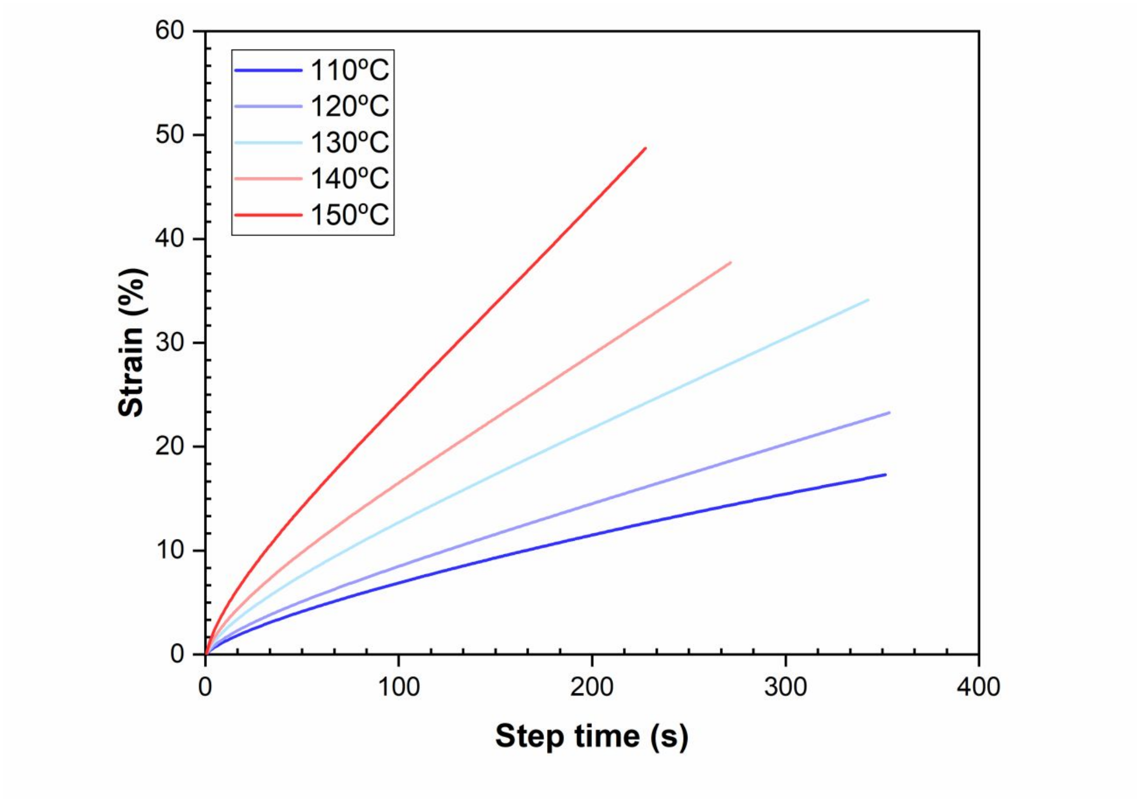

Figure S27 Creep curves of formulation Gly-Van-Im/Cyst at temperatures from 110 °C to 150 °C.

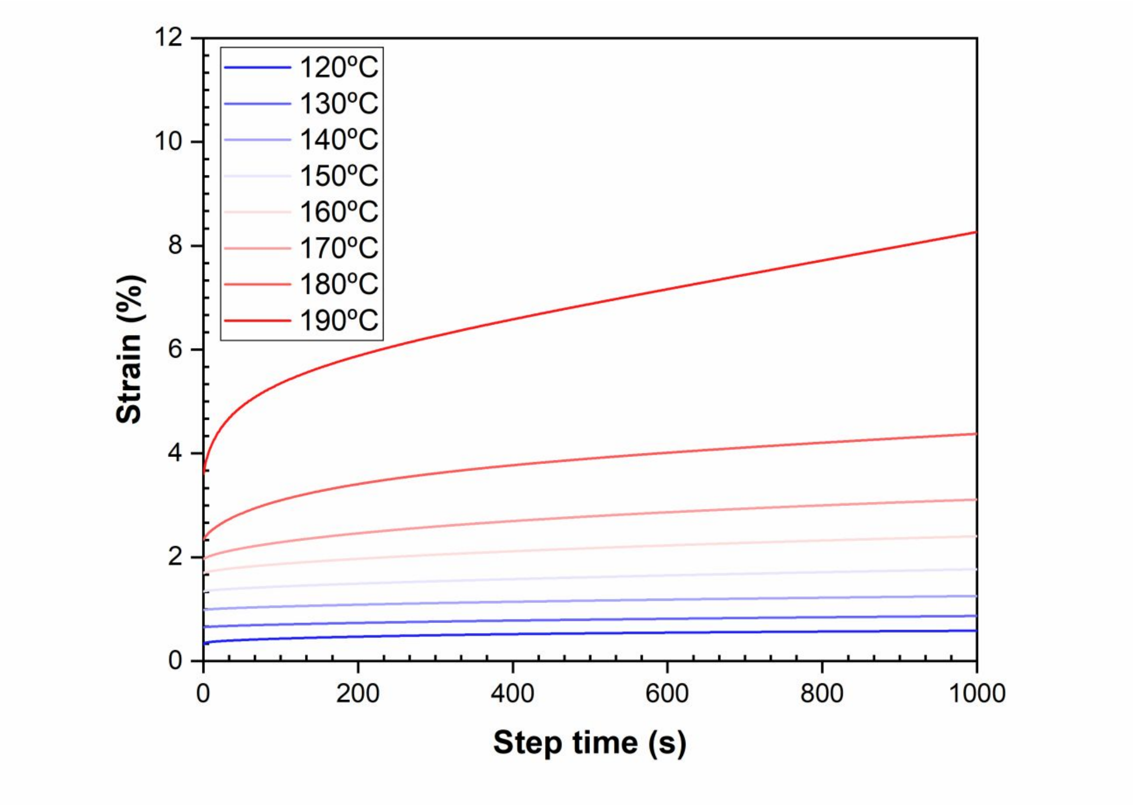

Figure S28 Creep curves of formulation DGEBA/Cyst at temperatures from 120 °C to 190 °C.

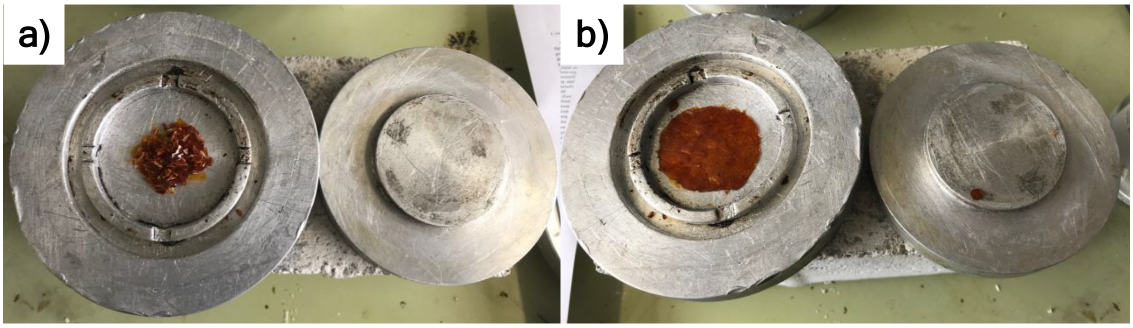

Figure S29 Gly-Van-Im/Cyst material (a) cut into small pieces and (b) after mechanical recycling through hot-pressing.
